# Admixture mapping reveals evidence of differential multiple sclerosis risk by genetic ancestry

**DOI:** 10.1371/journal.pgen.1007808

**Published:** 2019-01-17

**Authors:** Calvin Chi, Xiaorong Shao, Brooke Rhead, Edlin Gonzales, Jessica B. Smith, Anny H. Xiang, Jennifer Graves, Amy Waldman, Timothy Lotze, Teri Schreiner, Bianca Weinstock-Guttman, Gregory Aaen, Jan-Mendelt Tillema, Jayne Ness, Meghan Candee, Lauren Krupp, Mark Gorman, Leslie Benson, Tanuja Chitnis, Soe Mar, Anita Belman, Theron Charles Casper, John Rose, Manikum Moodley, Mary Rensel, Moses Rodriguez, Benjamin Greenberg, Llana Kahn, Jennifer Rubin, Catherine Schaefer, Emmanuelle Waubant, Annette Langer-Gould, Lisa F. Barcellos

**Affiliations:** 1 Genetic Epidemiology and Genomics Laboratory, University of California, Berkeley, Berkeley, California, United States of America; 2 Computational Biology Graduate Group, University of California, Berkeley, Berkeley, California, United States of America; 3 Department of Research & Evaluation, Kaiser Permanente Southern California, Los Angeles, California, United States of America; 4 Department of Neurology, University of California, San Francisco, San Francisco, California, United States of America; 5 Leukodystrophy Center, Children’s Hospital of Philadelphia, Philadelphia, Pennsylvania, United States of America; 6 Neurology and Developmental Neuroscience Department, Texas Children’s Hospital, Houston, Texas, United States of America; 7 University of Colorado School of Medicine, Aurora, Colorado, United States of America; 8 Department of Neurology, State University of New York, Buffalo, Buffalo, New York, United States of America; 9 Loma Linda University, Loma Linda, California, United States of America; 10 Department of Neurology, Mayo Clinic, Rochester, Minnesota, United States of America; 11 Children’s of Alabama, Birmingham, Alabama, United States of America; 12 Department of Pediatrics, University of Utah, Salt Lake City, Utah, United States of America; 13 Department of Neurology, NYU Langone Health, New York, New York, United States of America; 14 Boston Children’s Hospital, Boston, Massachusetts, United States of America; 15 MassGeneral Hospital for Children, Massachusetts General Hospital, Boston, Massachusetts, United States of America; 16 Department of Neurology, Washington University School of Medicine in St. Louis, St. Louis, Missouri, United States of America; 17 Department of Neurology, University of Utah, Salt Lake City, Utah, United States of America; 18 Center for Pediatric Neurosciences, Cleveland Clinic, Cleveland, Ohio, United States of America; 19 Mellen Center, Cleveland Clinic, Cleveland, Ohio, United States of America; 20 Neurology & Neurotherapeutics, University of Texas Southwestern, Dallas, Texas, United States of America; 21 Children’s National Medical Center, Northwest Washington, D.C., United States of America; 22 Ann & Robert H. Lurie Children’s Hospital of Chicago, Chicago, Illinois, United States of America; 23 Kaiser Permanente Division of Research, Kaiser Permanente Northern California, Oakland, California, United States of America; 24 Kaiser Permanente, Southern California Permanente Medical Group, Pasadena, California, United States of America; 25 Los Angeles Medical Center, Neurology Department, Los Angeles, California, United States of America; Case Western Reserve University School of Medicine, UNITED STATES

## Abstract

Multiple sclerosis (MS) is an autoimmune disease with high prevalence among populations of northern European ancestry. Past studies have shown that exposure to ultraviolet radiation could explain the difference in MS prevalence across the globe. In this study, we investigate whether the difference in MS prevalence could be explained by European genetic risk factors. We characterized the ancestry of MS-associated alleles using RFMix, a conditional random field parameterized by random forests, to estimate their local ancestry in the largest assembled admixed population to date, with 3,692 African Americans, 4,915 Asian Americans, and 3,777 Hispanics. The majority of MS-associated human leukocyte antigen (HLA) alleles, including the prominent HLA-DRB1*15:*01* risk allele, exhibited cosmopolitan ancestry. Ancestry-specific MS-associated HLA alleles were also identified. Analysis of the HLA-DRB1*15:*01* risk allele in African Americans revealed that alleles on the European haplotype conferred three times the disease risk compared to those on the African haplotype. Furthermore, we found evidence that the European and African HLA-DRB1*15:*01* alleles exhibit single nucleotide polymorphism (SNP) differences in regions encoding the HLA-DRB1 antigen-binding heterodimer. Additional evidence for increased risk of MS conferred by the European haplotype were found for HLA-B*07:*02* and HLA-A*03:*01* in African Americans. Most of the 200 non-HLA MS SNPs previously established in European populations were not significantly associated with MS in admixed populations, nor were they ancestrally more European in cases compared to controls. Lastly, a genome-wide search of association between European ancestry and MS revealed a region of interest close to the *ZNF596* gene on chromosome 8 in Hispanics; cases had a significantly higher proportion of European ancestry compared to controls. In conclusion, our study established that the genetic ancestry of MS-associated alleles is complex and implicated that difference in MS prevalence could be explained by the ancestry of MS-associated alleles.

## Author summary

Multiple sclerosis (MS) is an autoimmune disease that is mostly found in populations with northern European ancestry. Our study investigates whether there is evidence that the difference in MS prevalence around the globe could be explained by European MS genetic risk factors in African Americans, Hispanics, or Asian Americans. Our work first established that most alleles associated with MS are not necessarily European, but are in fact, cosmopolitan. However, we also observed in African Americans that European HLA-DRB1*15:*01* conferred three times the odds of MS compared to the African allele. The allele HLA-DRB1*15:*01* has been shown to be a major genetic risk factor in Europeans and other admixed populations. In addition, we observed genetic variations between European and African HLA-DRB1*15:*01* alleles that, based on location, could influence the function of antigen-binding proteins involved in MS. Consequently, it is plausible that ancestry could explain the risk or protective effects of other MS-associated alleles. Additionally, our study found on chromosome 8 in Hispanics a region where MS cases have more European ancestry than controls, implicating there may be new MS risk alleles to be discovered in Hispanics. In conclusion, our study found evidence that the difference in MS prevalence could be explained by European ancestry and established that the ancestry of MS genetic risk factors is complex.

## Introduction

Multiple sclerosis (MS) is an autoimmune disease of the central nervous system that results in demyelination and tissue loss. Association studies in White, non-Hispanic populations have discovered human leukocyte antigen (HLA) alleles conferring strong risk and protective effects and 200 non-HLA genetic risk variants conferring modest risk of MS[1,2]. Evidence that HLA class II alleles interact to confer greater risk of MS have been found[3]. Together, identified MS genetic risk factors are estimated to explain up to 30% of total heritability, of which most is accounted for by HLA alleles[2,4].

The prevalence of MS varies across the globe but is highest in White, non-Hispanic populations. There is evidence that African Americans are at higher risk for developing the disease, and along with Hispanics, may have a more severe disease course. Incidentally, countries with majority White, non-Hispanic individuals and experience highest MS prevalence are located at higher latitudes. Past studies have not only established the association between ultraviolet radiation and MS prevalence, but have also found evidence supporting the causal role of low vitamin D on MS risk. In this study, we investigate another hypothesis—that the difference in MS prevalence across the globe can be explained by European ancestry. If European ancestry can explain this difference, then MS-associated alleles in admixed individuals can either be European or confer increased risk on a European haplotype compared to a non-European haplotype.

We investigate this by analyzing the genetic ancestry of MS-associated alleles in a large combined cohort totaling 1,471 MS cases and 10,913 controls including African American, Asian American, and Hispanic individuals. Previous studies have been able to replicate the association of the HLA risk allele HLA-DRB1*15:*01* in nearly all populations[5]. Additional HLA alleles have been found to be associated with MS in non-European populations, such as HLA-DRB1*15:*03* in African Americans and HLA-DRB1*04:*05* in the Japanese population[6]. Limited replication has been achieved for non-HLA genetic risk variants in other populations[7–9]. We found that most MS-associated alleles are cosmopolitan, but there is evidence that European risk alleles may confer more risk than non-European risk alleles, most notably for the major risk allele HLA-DRB1*15:*01*. Thus, there is evidence that the difference in MS prevalence could be explained by European ancestry. We also tested for the association of European ancestry with MS across the genome in African Americans, Asian Americans and Hispanics, and found a signal of association on chromosome 8 in Hispanics.

## Results

### Analysis of population structure

**Fig 1:**
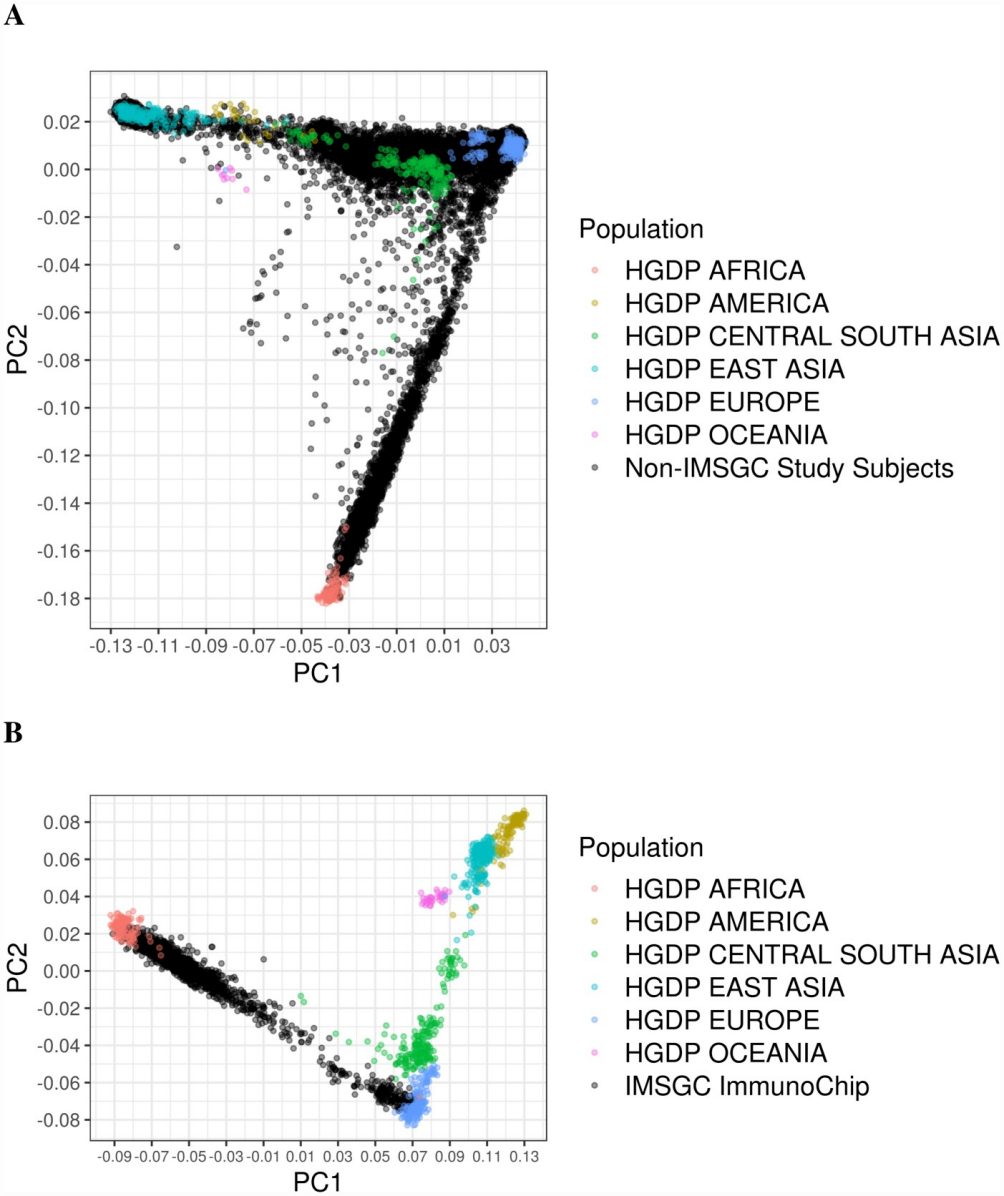
Multidimensional scaling analysis of study subjects with HGDP reference samples. (A) Study subjects from Northern California Kaiser Permanente, Southern California Kaiser Permanente, U.S. Pediatric MS Network, Genetic Epidemiology Research on Aging datasets, and (B) IMSGC Immunochip.

We performed multidimensional scaling (MDS) analysis on genotype data from 21,647 subjects to generate components used to control for population stratification in later analyses (Fig 1A, S19 Table). This analysis was done separately for African American samples which were genotyped using the Illumina Immunochip (Fig 1B, S19 Table). The first three components were sufficient to differentiate global ancestries and broadly categorize samples as African Americans, Asian Americans, or Hispanics. Component 2 was correlated with African ancestry in African Americans (R = 1.00, p < 0.01), component 1 was correlated with Native American ancestry in Hispanics (R = -0.95, p < 0.01), and component 1 was correlated with East Asian ancestry in Asian Americans (R = 0.99, p < 0.01).

We used fastSTRUCTURE to estimate global admixture proportions for individuals from each admixed population. After eliminating White, non-Hispanic individuals and Hispanics with less than 5% Native American ancestry, a total of 3,692 African Americans, 4,915 Asian Americans, and 3,777 Hispanics comprised the final dataset (Table 1). African Americans were estimated to be 76.1% African and 23.9% European on average, Asian Americans were estimated to be 92.2% East Asian and 7.8% European on average, and Hispanics were estimated to be 68.4% European, 28.8% Native American, and 2.8% African on average, in line with published estimates[10].

**Table 1:** Number of cases and controls by admixed population.

Population	Case (n)	Control (n)
African Americans	1,081	2,611
Hispanics	326	3,451
Asian Americans	64	4,851

Number of MS cases and controls for African American, Hispanic, and Asian American datasets, after removing related individuals ($\hat{\pi }>0.25$), White, non-Hispanic subjects, and Hispanics with less than 5% Native American ancestry. n = number of individuals.

**Fig 2:**
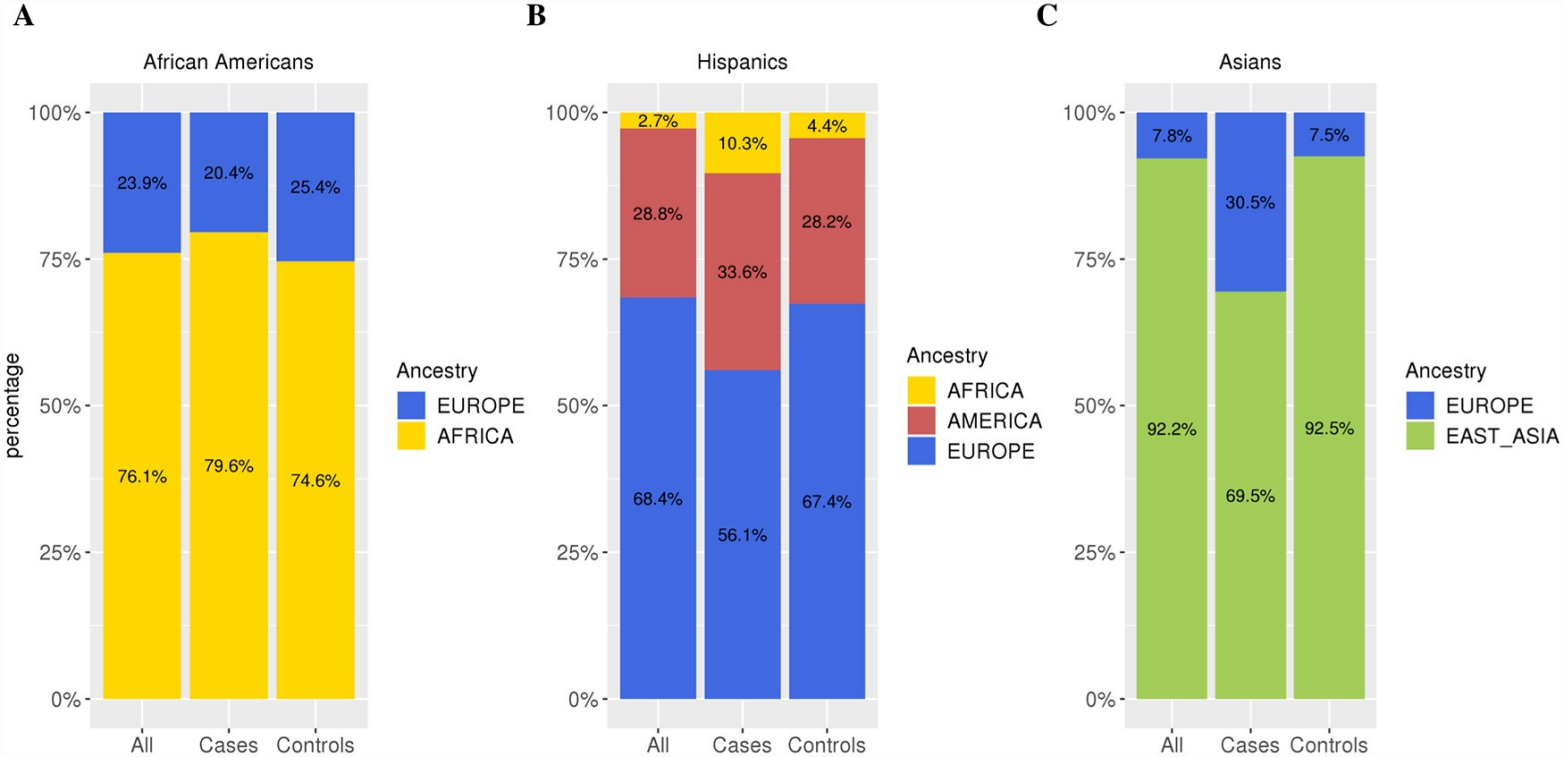
Global admixture proportions of study subjects. Global admixture proportion estimates by fastSTRUCTURE with HGDP reference samples. Proportions are shown by case/control status and with cases and controls combined for (A) African Americans, (B) Hispanics, and (C) Asian Americans. The x-axis label ‘All’ denotes admixture proportions for cases and controls combined. See Table 1 for the sample numbers corresponding to each admixed population.

The average global admixture for MS cases and controls is shown in Fig 2 (S20 Table). We observed significant differences in global admixture proportions between cases and controls across all populations. African American cases had 5.0% increased African ancestry compared to controls (p < 0.01); Hispanic cases had 5.4% increased Native American ancestry (p = 0.02) and 11.3% decreased European ancestry (p < 0.01) compared to controls. Asian American cases had 23.0% increased European ancestry compared to controls (p < 0.01).

### Ancestry association at the MHC

**Fig 3:**
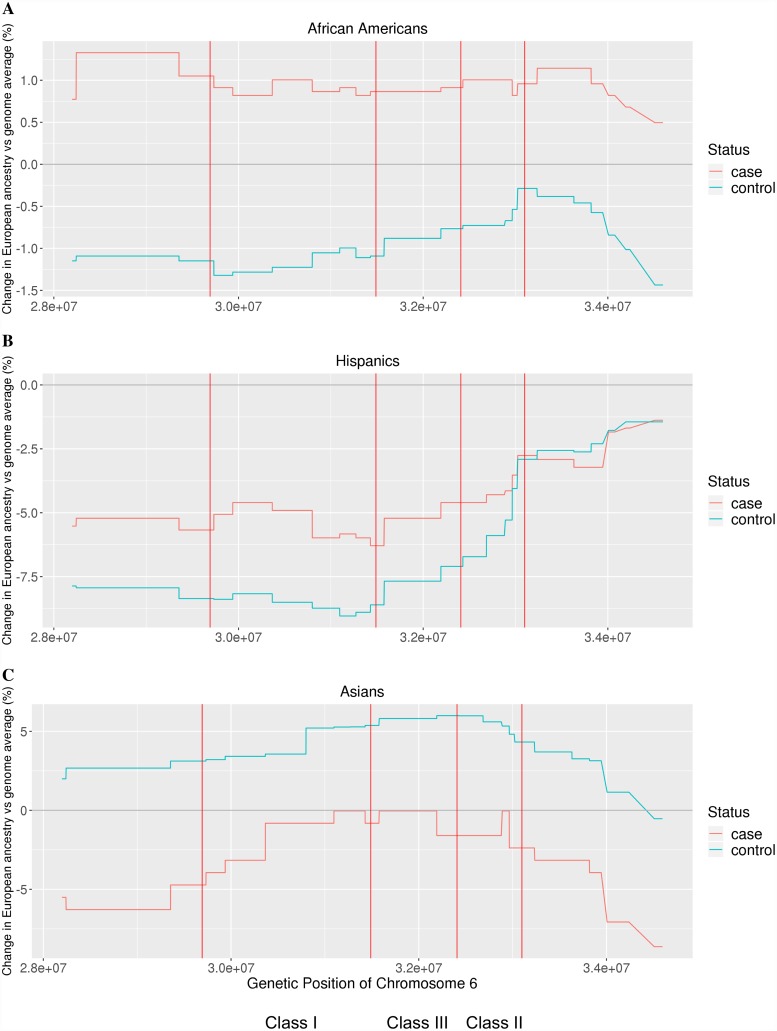
Deviation of local from global European ancestry. The difference between local and global ancestry at the major histocompatibility complex (MHC) region, plotted for cases and controls. The red vertical bars denote borders of class I, II, and III of the MHC. Local and global ancestries are estimated with RFMix. For both (A) African Americans and (B) Hispanics, cases tended to have higher European ancestry than controls at the MHC. For (C) Asian Americans, cases tended to have lower European ancestry than controls at the MHC.

In previous studies, up to eleven regions within the major histocompatibility complex (MHC) have been identified to exhibit statistically significant independent effects of association with MS in White, non-Hispanic populations: six *HLA-DRB1*, one *HLA-DPB1*, one *HLA-A*, two *HLA-B* alleles, and one signal in a region spanning from *MICB* to *LST1*[11]. We tested each of these regions, in addition to regions spanned by *DQB1* and *HLA-C* and the broader regions class I, II, and III, for evidence of increased European ancestry in MS cases compared to controls. Results are summarized in Tables 2–4 and shown in Fig 3 (S21 Table). In African Americans, cases exhibited increased European ancestry at the MHC region compared to controls, after accounting for global admixture proportion differences, with genes in the class I region and the *MICB-LST1* region reaching statistical significance (p < 0.05). In Hispanics, the direction of association was the same as in African Americans, but none of the regions reached statistical significance. In Asian Americans, the cases had decreased European ancestry at the MHC region compared to controls, with the regions *HLA-DQB1* and *HLA-DRB1* demonstrating evidence for statistical significance (p < 0.05).

**Table 2:** European ancestry association with MS at regions of the MHC in African Americans.

MHC Region	${\overset{-}{z}}_{l,\;d}(k)-\;{\overset{-}{q}}_{d}(k)$	${\overset{-}{z}}_{l,c}(k)-\;{\overset{-}{q}}_{c}(k)$	z score	P value
*HLA A*	9.12E-3	-1.32E-2	2.04	2.06E-2
Class I	8.78E-3	-1.19E-2	1.91	2.83E-2
*MICB-LST1*	8.66E-3	-1.09E-2	1.78	3.79E-2
*HLA B*	8.19E-3	-1.11E-2	1.75	4.05E-2
*HLA C*	9.12E-3	-9.96E-3	1.72	4.24E-2
Class III	8.84E-3	-8.59E-3	1.59	5.57E-2
*DQB1*	1.00E-3	-7.28E-3	1.57	5.80E-2
*DRB1*	1.00E-3	-7.28E-3	1.57	5.80E-2
Class II	9.70E-3	-6.27E-3	1.47	7.10E-2
*DPB1*	9.58E-3	-2.87E-3	1.13	1.30E-1

Tests of European ancestry association with MS using test statistic for admixture mapping, sorted by P value. The column ${\overset{-}{z}}_{l,\;d}(k)-\;{\overset{-}{q}}_{d}(k)$ represents difference in average local and global European ancestry (*k*) proportions for cases *d*. The column ${\overset{-}{z}}_{l,\;c}(k)-\;{\overset{-}{q}}_{c}(k)$ is defined similarly as ${\overset{-}{z}}_{l,\;d}(k)-\;{\overset{-}{q}}_{d}(k)$ for controls *c*. The z score is the admixture mapping test statistic calculated as described in Materials and Methods. Adj P value is Bonferroni-adjusted p value.

**Table 3:** European ancestry association with MS at regions of the MHC in Hispanics.

MHC Region	${\overset{-}{z}}_{l,\;d}(k)-\;{\overset{-}{q}}_{d}(k)$	${\overset{-}{z}}_{l,c}(k)-\;{\overset{-}{q}}_{c}(k)$	z score	P value
Class I	-5.18E-2	-8.48E-2	1.57	5.76E-2
*HLA A*	-5.06E-2	-8.39E-2	1.55	6.11E-2
*HLA C*	-5.83E-2	-9.04E-2	1.51	6.51E-2
*HLA B*	-5.98E-2	-8.89E-2	1.38	8.43E-2
Class III	-5.09E-2	-7.55E-2	1.22	1.12E-1
*MICB-LST1*	-6.29E-2	-8.60E-2	1.11	1.34E-1
*DQB1*	-4.60E-2	-6.72E-2	1.02	1.54E-1
*DRB1*	-4.60E-2	-6.72E-2	1.02	1.54E-1
Class II	-4.00E-2	-5.32E-2	0.66	2.54E-1
*DPB1*	-2.76E-2	-2.91E-2	0.07	4.71E-1

Tests of European ancestry association with MS using test statistic for admixture mapping, sorted by P value. The column ${\overset{-}{z}}_{l,\;d}(k)-\;{\overset{-}{q}}_{d}(k)$ represents difference in average local and global European (*k*) ancestry proportions for cases *d*. The quantity ${\overset{-}{z}}_{l,\;c}(k)-\;{\overset{-}{q}}_{c}(k)$ is defined similarly for controls *c*. The z score is the admixture mapping test statistic calculated as described in Materials and Methods.

**Table 4:** European ancestry association with MS at regions of the MHC in Asian Americans.

MHC Region	${\overset{-}{z}}_{l,\;d}(k)-\;{\overset{-}{q}}_{d}(k)$	${\overset{-}{z}}_{l,c}(k)-\;{\overset{-}{q}}_{c}(k)$	z score	P value
*DQB1*	-1.60E-2	5.98E-2	-1.67	4.80E-2
*DRB1*	-1.60E-2	5.98E-2	-1.67	4.80E-2
*HLA A*	-3.94E-2	3.21E-2	-1.56	5.94E-2
Class II	-1.67E-2	5.35E-2	-1.52	6.39E-2
Class III	-7.52E-3	5.83E-2	-1.48	7.00E-2
*DPB1*	-2.38E-2	4.32E-2	-1.43	7.69E-2
*MICB-LST1*	-8.18E-3	5.37E-2	-1.36	8.62E-2
Class I	-1.90E-2	4.07E-2	-1.33	9.24E-2
*HLA B*	-3.69E-4	5.28E-2	-1.14	1.27E-1
*HLA C*	-3.69E-4	5.27E-2	-1.14	1.27E-1

Tests of European ancestry association with MS using test statistic for admixture mapping, sorted by P value. The column ${\overset{-}{z}}_{l,\;d}(k)-\;{\overset{-}{q}}_{d}(k)$ represents difference in average local and global European (*k*) ancestry proportions for cases *d*. The quantity ${\overset{-}{z}}_{l,\;c}(k)-\;{\overset{-}{q}}_{c}(k)$ is defined similarly for controls *c*. The z score is the admixture mapping test statistic calculated as described in Materials and Methods.

### Ancestry of MS-associated HLA alleles

**Fig 4:**
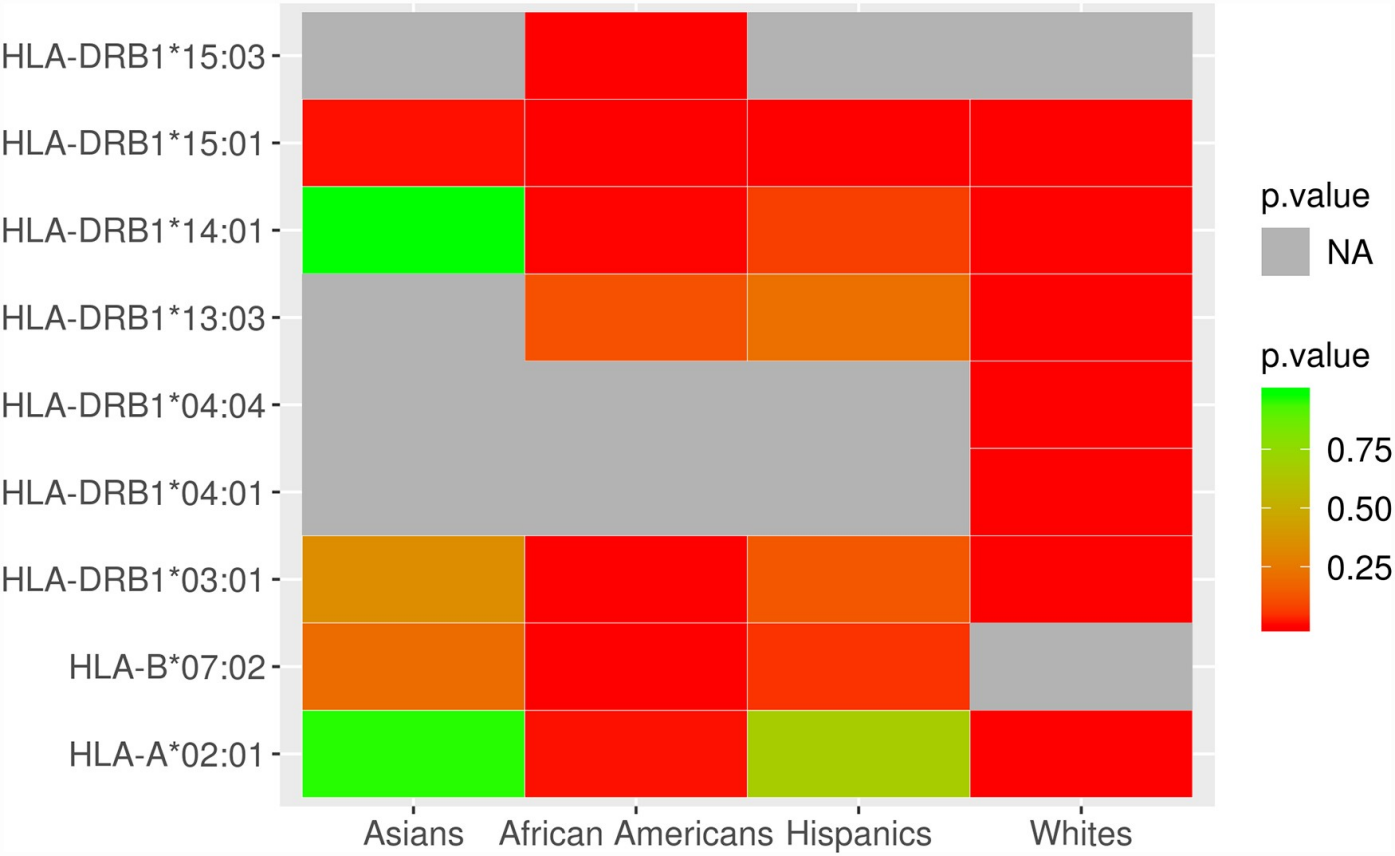
Comparison of MS-associated HLA alleles across populations. P-value heat map for HLA alleles that reached Bonferroni significance in either Asian Americans, African Americans, Hispanics, or White, non-Hispanic individuals. P-values of HLA alleles associated with MS in White, non-Hispanic individuals were taken from previous work[11]. The HLA allele HLA-DRB1*15:*01* was most consistently associated with MS across all four populations, followed by HLA-DRB1*03:*01*. Gray (NA) denotes an HLA allele that is missing due to not being present in the population or failed to pass HLA imputation QC.

We investigated the ancestry of MS-associated HLA alleles to determine whether ancestry associations observed at the regions within the MHC could be explained. We first identified HLA alleles associated with MS in each admixed group using additive multivariate logistic regression, adjusting for the first three MDS components. We observed 14 alleles in African Americans, 15 alleles in Hispanics, and 4 alleles in Asian Americans that reached nominal significance of association (p < 0.05). HLA-DRB1*15:*01*, the strongest genetic association with MS observed in White, non-Hispanic individuals, to date, was a top signal across all three admixed populations, consistent with previous findings[5]. As expected, the African allele HLA-DRB1*15:*03* was significantly associated with MS in African Americans[12]. In African Americans, we further replicated the association of HLA risk alleles previously established in the White, non-Hispanic population: HLA-DRB1*03:*01*, HLA-A*02:*01*, HLA-DRB1*14:*01*, and HLA-B*38:*01* at nominal level significance (p < 0.05)[11]. In both Hispanics and Asian Americans, HLA-DRB1*15:*01* is the only established HLA risk alleles in White, non-Hispanics that was replicated. Fig 4 (S22 Table) compares the p-values of significant MS-associated HLA alleles across different populations. With our sample sizes, we estimate close to 100% power of detection for African Americans and Hispanics and 80% power for Asian Americans. Assuming the MS HLA alleles found in the European population are also associated with MS in admixed populations, then 6, 7, and 4 HLA alleles are expected to be detected in African Americans, Hispanics, and Asian Americans respectively, post quality control.

**Fig 5:**
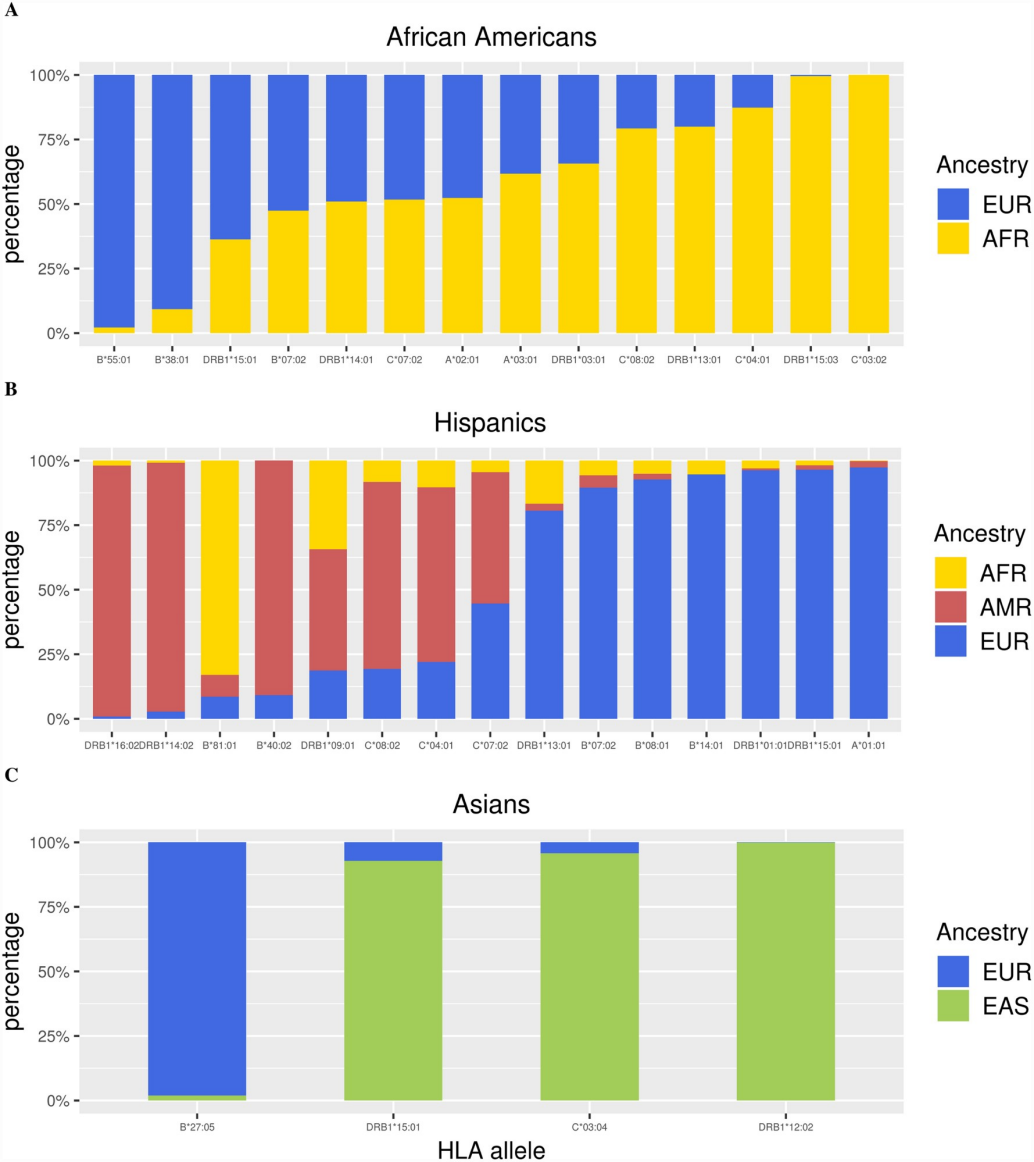
Admixture of HLA alleles associated with MS. Ancestry was inferred for MS-associated HLA alleles that passed QC using RFMix. MS-associated alleles were significant at the nominal level (p < 0.05), had imputation score R^2^ > 0.80, and had minor allele frequency greater than 0.005. Other than HLA-B*55:*01* in (A) African Americans, HLA-DRB1*15:*01*, HLA-DRB1*16:*02*, HLA-DRB1*01:*01*, HLA-DRB1*14:*02*, HLA-A*01:*01* in (B) Hispanics, and HLA-B*27:*05* and HLA-C*03:*04* in (C) Asian Americans, HLA alleles associated with MS are cosmopolitan.

Next, we estimated the admixture proportions of all the nominally-associated alleles using local ancestry estimates from RFMix. Analysis of HLA alleles and corresponding admixture proportions are shown in Tables 5–7, and Fig 5 (S23 Table). Ancestry estimates for HLA alleles previously established to be ancestry-specific were in strong agreement: 98.4% East Asian for the East Asian allele HLA-DRB1*04:*05* in Asian Americans (n = 692 alleles), 96.2% European for the European allele HLA-DRB1*01:*01* in Hispanics (n = 395 alleles), 96.4% Native American for Native American allele HLA-DRB1*14:*02* in Hispanics (n = 454 alleles), and 99.5% African for African allele HLA-DRB1*15:*03* in African Americans(n = 881 alleles)[13]. Most MS-associated HLA alleles are cosmopolitan across the admixed populations. The MS risk allele HLA-DRB1*15:*01*, which is more common in Europeans, was estimated to be 63.7% European in African Americans (n = 512 alleles) and 96.4% European in Hispanics (n = 534 alleles)[14]. However, it is striking that HLA-DRB1*15:*01* is 92.9% East Asian in Asian Americans (n = 1,228 alleles).

**Table 5:** Ancestry of HLA alleles associated with MS in African Americans.

Allele	N	OR	P value	Adj P value	EUR	AFR
HLA-DRB1*15:*01*	512	2.00 (1.58–2.55)	1.26E-8	8.83E-7	0.64	0.36
HLA-DRB1*03:*01*	658	1.45 (1.22–1.72)	2.61E-5	1.83E-3	0.34	0.66
HLA-B*07:*02*	598	1.48 (1.23–1.78)	3.61E-5	2.53E-3	0.53	0.47
HLA-DRB1*15:*03*	881	1.37 (1.17–1.59)	8.11E-5	5.68E-3	0.00	1.00
HLA-DRB1*14:*01*	106	0.39 (0.22–0.70)	1.37E-3	9.58E-2	0.49	0.51
HLA-A*03:*01*	694	1.3 (1.10–1.54)	1.97E-3	1.38E-1	0.38	0.62
HLA-A*02:*01*	914	0.8 (0.68–0.94)	7.88E-3	5.51E-1	0.48	0.52
HLA-C*08:*02*	198	1.44 (1.07–1.94)	1.47E-2	1.00E+0	0.21	0.79
HLA-B*55:*01*	45	0.24 (0.07–0.78)	1.74E-2	1.00E+0	0.98	0.02
HLA-DRB1*13:*01*	476	0.76 (0.60–0.96)	2.09E-2	1.00E+0	0.20	0.80
HLA-C*04:*01*	1704	0.87 (0.76–0.98)	2.35E-2	1.00E+0	0.13	0.87
HLA-C*07:*02*	708	1.22 (1.02–1.45)	2.55E-2	1.00E+0	0.48	0.52
HLA-B*38:*01*	43	0.28 (0.09–0.94)	3.87E-2	1.00E+0	0.91	0.09
HLA-C*03:*02*	95	0.57 (0.33–0.98)	4.30E-2	1.00E+0	0.00	1.00

HLA alleles that were nominally associated with MS (p < 0.05) and their ancestry proportions estimated from RFMix. Odds ratio (OR) of association for case-control comparison are also shown along with their 95% confidence interval. All tested HLA alleles passed imputation quality score (R^2^ > 0.80) and have allele frequencies greater than 0.005. N = number of alleles; EUR = European; AFR = African; Adj P value = Bonferroni adjusted p-value.

**Table 6:** Ancestry of HLA alleles associated with MS in Hispanics.

Allele	N	OR	P value	Adj P value	EUR	AMR	AFR
HLA-DRB1*15:*01*	534	2.45 (1.88–3.19)	2.59E-11	2.00E-9	0.96	0.02	0.02
HLA-DRB1*16:*02*	180	1.92 (1.23–2.99)	4.11E-3	3.17E-1	0.01	0.97	0.02
HLA-DRB1*13:*01*	314	0.47 (0.27–0.84)	1.13E-2	8.66E-1	0.81	0.03	0.17
HLA-C*04:*01*	1601	0.76 (0.61–0.95)	1.52E-2	1.00E+0	0.22	0.68	0.1
HLA-DRB1*01:*01*	395	0.54 (0.33–0.89)	1.65E-2	1.00E+0	0.96	0.01	0.03
HLA-B*08:*01*	318	1.54 (1.08–2.21)	1.71E-2	1.00E+0	0.93	0.02	0.05
HLA-B*40:*02*	545	1.39 (1.05–1.84)	2.22E-2	1.00E+0	0.09	0.91	0.00
HLA-DRB1*14:*02*	454	0.60 (0.39–0.94)	2.49E-2	1.00E+0	0.03	0.96	0.01
HLA-C*08:*02*	327	1.52 (1.05–2.20)	2.53E-2	1.00E+0	0.19	0.72	0.08
HLA-DRB1*09:*01*	160	0.39 (0.17–0.91)	2.90E-2	1.00E+0	0.19	0.47	0.34
HLA-A*01:*01*	521	1.40 (1.03–1.91)	3.29E-2	1.00E+0	0.97	0.02	0.00
HLA-C*07:*02*	976	1.28 (1.02–1.62)	3.55E-2	1.00E+0	0.45	0.51	0.05
HLA-B*14:*01*	56	2.16 (1.03–4.55)	4.26E-2	1.00E+0	0.95	0.00	0.05
HLA-B*81:*01*	47	2.38 (1.02–5.54)	4.44E-2	1.00E+0	0.09	0.09	0.83
HLA-B*07:*02*	460	1.36 (1.00–1.85)	4.86E-2	1.00E+0	0.90	0.05	0.06

HLA alleles that were nominally associated with MS (p < 0.05) and their ancestry proportions estimated from RFMix. Odds ratio (OR) of association for case-control comparison are also shown along with their 95% confidence interval. All tested HLA alleles passed imputation quality score (R^2^ > 0.80) and had allele frequencies greater than 0.005. N = number of alleles; EUR = European; AMR = American; AFR = African. Adj P value = Bonferroni adjusted p-value.

**Table 7:** Ancestry of HLA alleles associated with MS in Asian Americans.

Allele	N	OR	P value	Adj P value	EUR	EAS
HLA-B*27:*05*	50	6.74 (1.92–23.66)	2.92E-3	1.34E-1	0.98	0.02
HLA-DRB1*15:*01*	1228	1.88 (1.19–2.99)	7.26E-3	3.34E-1	0.07	0.93
HLA-C*03:*04*	1687	1.69 (1.04–2.76)	3.43E-2	1.00E+0	0.04	0.96
HLA-DRB1*12:*02*	1139	0.32 (0.11–0.94)	3.75E-2	1.00E+0	0.00	1.00

HLA alleles that were nominally associated with MS (p < 0.05) and their ancestry proportions estimated from RFMix. Odds ratio (OR) of association for case-control comparison are also shown along with their 95% confidence interval. All tested HLA alleles passed imputation quality score (R^2^ > 0.80) and had allele frequencies greater than 0.005. N = number of alleles; EUR = European; EAS = East Asian. Adj P value = Bonferroni adjusted p-value.

We searched for MS-associated HLA alleles that are potentially ancestry-specific, imposing a 96% ancestry cutoff because we were able to correctly estimate the ancestry of HLA alleles of known ancestry as 96% or greater. Briefly, we considered an MS-associated allele as a candidate ancestry-specific allele if at least 96% of its ancestry comes from a single ancestry across all admixed populations in which it exists, and/or is missing in the rest of admixed populations. An allele could be missing because it does not exist in other ancestries (e.g. African HLA-DRB1*15:*03*), or because it did not pass quality control for imputation. Using this approach, we classified HLA-DRB1*14:*02* and HLA-DRB1*16:*02* as Native American alleles, HLA-DRB1*15:*03* as an African risk allele, HLA-DRB1*12:*02* as an East Asian allele, and HLA-B*55:*01*, HLA-B*27:*05*, and HLA-A*01:*01* as European alleles.

### Risk of MS between European and African HLA alleles in African Americans

Given that African Americans exhibit two-way admixture and many MS-associated HLA alleles in African Americans are relatively admixed, we studied the differential risk of HLA alleles in African Americans based on ancestry. We first performed a case-control study of the prominent MS risk allele HLA-DRB1*15:*01* in African Americans to determine whether there were any differences in risk conferred by HLA-DRB1*15:*01* alleles of European and African origin. We removed 12 alleles from the analysis, of which 6 were from cases and 6 were from controls, whose HLA-DRB1*15:*01* allele was not inferred to be completely European or African. Table 8 shows the final number of alleles by ancestry and by case status. The risk of MS conferred by the European HLA-DRB1*15:*01* allele was determined from logistic regression to be three times higher compared to the African HLA-DRB1*15:*01* allele (OR = 3.00, 95% CI: 1.90–4.76, p = 2.49 × 10^−6^), after adjusting for the first 3 MDS components. We restricted the logistic regression to alleles from individuals with one copy of HLA-DRB1*15:*01* so that the association was not confounded by number of HLA-DRB1*15:*01* alleles.

**Table 8:** HLA-DRB1*15:*01* of European origin confers greater risk of MS compared to DRB1*15:*01* of African origin.

HLA-DRB1*15:*01* Ancestry	Case (n)	Control (n)	
European	129	191	319
African	43	137	180
	171	328	499

Two-by-two table of counts of HLA-DRB1*15:*01* alleles by ancestry and case/control status. The HLA-DRB1*15:*01* allele passed imputation quality score (R^2^ > 0.80) and had allele frequency greater than 0.005. Alleles that are not completely European or African were removed.

We continued the same analyses for other alleles in Table 5. Alleles with a sample size less than 50 or with a predominant ancestry of more than 90% are excluded from the analysis. This analysis further revealed that European HLA-B*07:*02* (OR = 1.66, 95% CI: 1.12–2.47, p = 1.18 × 10^−2^) and HLA-A*03:*01* (OR = 1.54, 95% CI: 1.04–2.29, p = 2.97 × 10^−2^) conferred a greater risk of MS compared to their African counterparts at p < 0.05. However, for the risk allele HLA-DRB1*03:*01*, the European allele is protective (OR = 0.64, 95% CI: 0.43–0.96, p = 3.03 × 10^−2^) compared to the African allele. Hence, this provides additional evidence that the European haplotype confers more risk of MS compared to the African haplotype for other HLA alleles, although this is not true for every allele (S1 Table).

**Fig 6:**
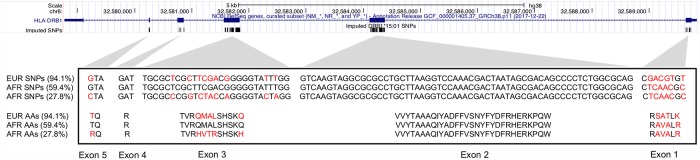
European and African HLA-DRB1*15:*01* subsequence comparison. Comparison of SNP and AA subsequences imputed by SNP2HLA for European and African HLA-DRB1*15:*01* alleles in African Americans. Note: subsequence implies the SNPs and AAs are not necessarily contiguous. The subsequences were aligned by position (GRCh38) with the UCSC genome browser NCBI gene track; the imputed positions correspond to exons 1–5 of *HLA-DRB1*. The top frequent (94.1%) SNP and amino acid subsequence for European HLA-DRB1*15:*01* was compared against the top two frequent (59.4% and 27.8%) subsequences for African HLA-DRB1*15:*01*. Red indicates a mismatch between any two given positions between an African and European allele. AA = amino acid; EUR = European; AFR = African.

SNP2HLA imputes SNPs and amino acids (AA) for the exons of HLA alleles, with a 1-to-1 mapping between a SNP and AA subsequence (see Methods). Given that European HLA-DRB1*15:*01* conferred three times the odds of MS compared to African HLA-DRB1*15:*01*, and without evidence that this finding was due to HLA-DQB1*06:*02* (S2 and S3 Tables), we compared the most representative SNP and AA subsequences for European and African HLA-DRB1*15:*01* alleles to look for differences. A large majority (94.1%) of European HLA-DRB1*15:*01* alleles shared the same SNP and AA subsequences, whereas African HLA-DRB1*15:*01* subsequences were more diverse. Data in S4–S7 Tables summarize the numbers of SNP and AA subsequences and S8 and S9 Tables show the genetic coordinates and AA positions for the imputation of *HLA-DRB1* subsequences. Fig 6 shows the comparison of the most frequent (94.1%) SNP and AA subsequence for European HLA-DRB1*15:*01* alleles against the top two most frequent (59.4% and 27.8% respectively) subsequences for African HLA-DRB1*15:*01* alleles, respectively. All differences between the European subsequence and the most frequent African subsequence were within exon 1. When compared against the second most frequent African subsequence, differences were found in exons 1, 3, and 6.

### Ancestry association at non-HLA MS genetic risk loci

We evaluated the association of European ancestry with MS for 200 established non-HLA genetic risk loci identified in White, non-Hispanic individuals. Following quality control (QC), 165 MS risk variants were available in African Americans, 167 MS risk variants in Hispanics, and 154 MS risk variants in Asian Americans for analysis. We tested each risk variant for association with MS and tested each genetic locus for association between European ancestry and MS. Data in S10–S12 Tables summarize the results for each admixed population, respectively. Increased East Asian ancestry in MS cases compared to controls for SNPs rs405343 (p = 5.53 × 10^−13^) and rs6670198 (p = 6.13 × 10^−8^) was observed in Asian Americans. No other genetic risk locus showed evidence of increased ancestry in cases compared to controls in any admixed population after adjustment for multiple tests. The risk allele T for SNP rs405343 was significantly associated with MS (OR = 2.55, 95% CI: 1.70–3.83, p = 6.87 x 10^−6^) in Asian Americans; however, the risk allele T for SNP rs6670198 showed no evidence for association. A small proportion of MS risk alleles overall demonstrated a nominal level of association at p < 0.05: 13 SNPs in African Americans, 21 SNPs in Hispanics, and 28 SNPs in Asian Americans. With our sample sizes, the powers of detection for African Americans, Hispanics, and Asians are estimated to be 21.5%, 26.5%, and 11.7%, respectively. Assuming the established MS non-HLA alleles are also associated with MS in admixed populations, then 35, 44, and 18 non-HLA alleles are expected to be detected in African Americans, Hispanics, and Asian Americans respectively, post quality control.

**Fig 7:**
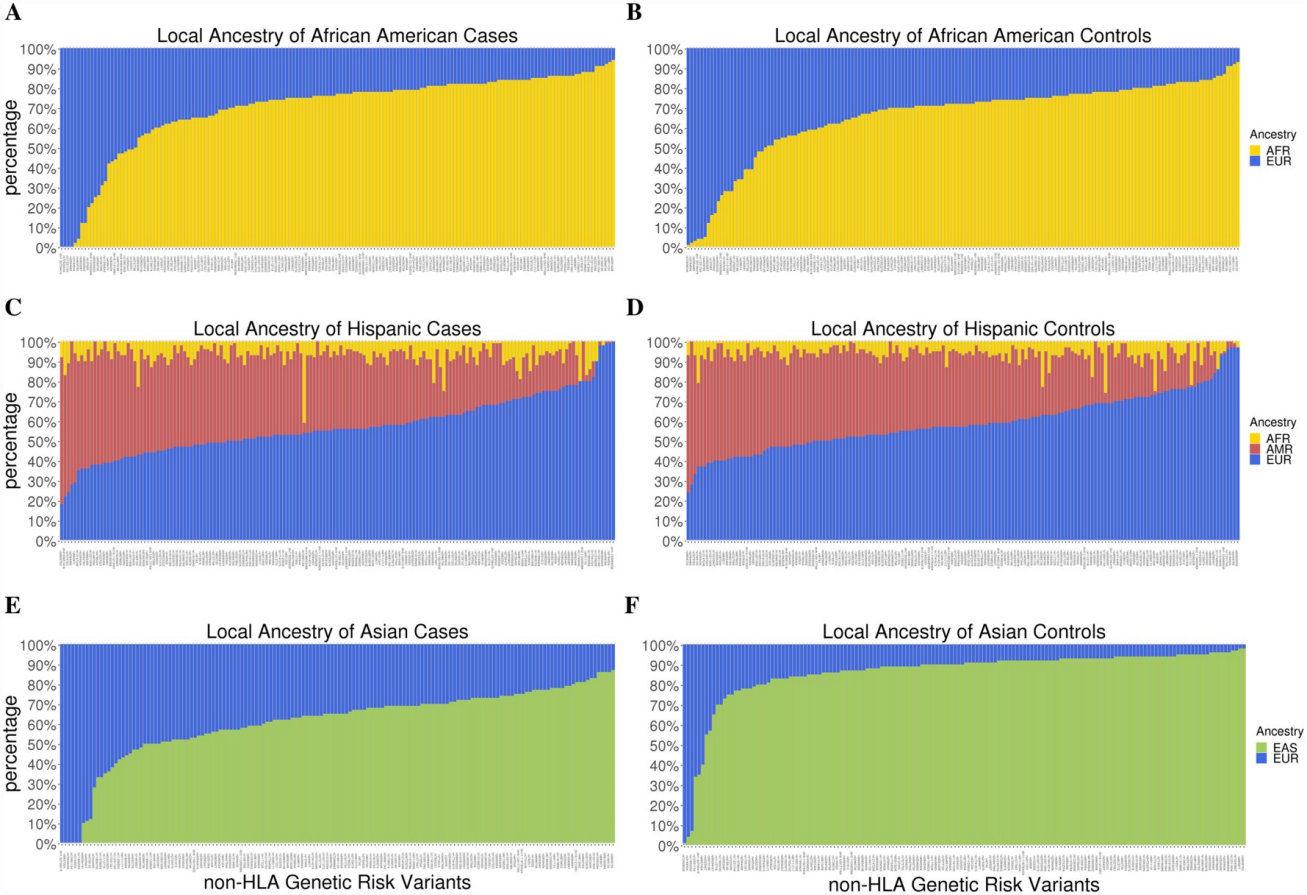
Admixture of non-HLA MS risk variants. Local ancestry estimates from RFMix for the non-HLA risk variants that passed QC, sorted in order of increasing European ancestry. The admixture proportions of risk variants were estimated separately in (A) African American cases, (B) African American controls, (C) Hispanic cases, (D) Hispanic controls, (E) Asian American cases, and (F) Asian American controls. The ancestry proportions of risk variants in cases and controls were largely reflective of global admixture proportions in cases and controls, respectively.

We determined whether European ancestry, both globally and locally at the non-HLA genetic risk loci, was correlated with a cumulative genetic risk score in African American, Asian American, and Hispanic MS cases. S1 Fig (S25 Table) shows results for each admixed population. Globally, no evidence for significant correlation was observed in African Americans (R = 0.04, p = 0.47), Hispanics (R = 0.06, p = 0.30), or Asian Americans (R = 0.25, p = 0.05); similar results were observed for local ancestry in all populations. Admixture estimates showed that the majority of the non-HLA variants investigated here were cosmopolitan; local admixture was reflective of global admixture patterns (Fig 7, S24 Table).

### Whole-genome association scan

**Fig 8:**
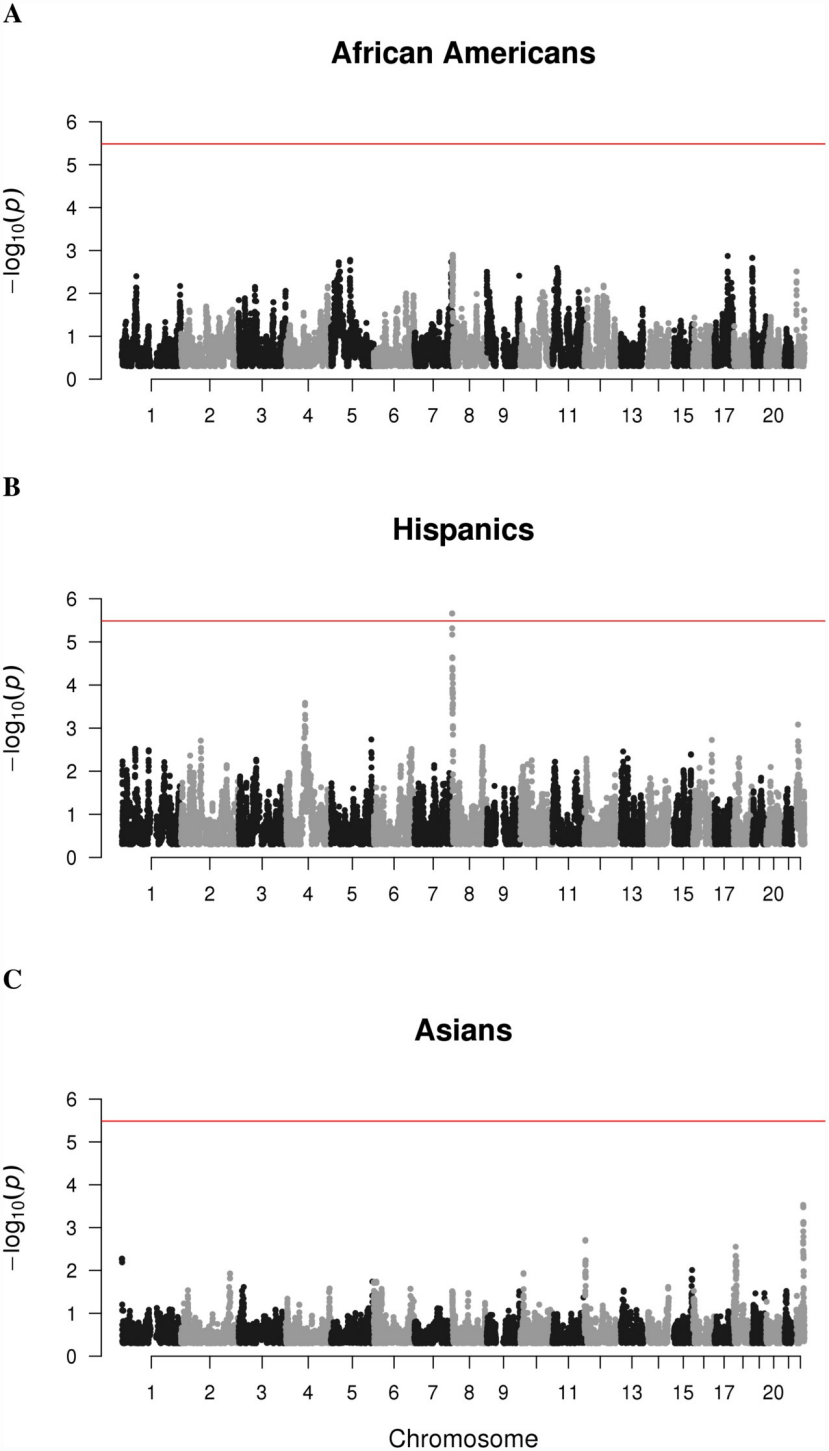
Genome-wide association of European ancestry with MS. P values from testing of association between European ancestry and MS using non-parametric test statistic proposed by Montana and Pritchard, as described in Methods. One locus was selected from each 0.2 cM window used by RFMix for ancestry inference to reduce the burden of multiple hypothesis testing, resulting in 15,282 tests. The red horizontal line indicates the negative log of the Bonferroni P value (p = 3.27 × 10^−6^) for establishing significance. (A) None of the loci tested for African Americans demonstrated evidence for significant association. (B) In Hispanics, a region spanning from 2Mb to 3Mb on chromosome 8 showed evidence for a significant association. (C) None of the loci tested for Asian Americans were significantly associated.

We searched across the genome in African Americans, Asian Americans, and Hispanics to identify regions where individuals with MS had a higher proportion of European ancestry compared to controls using the test statistic in Eq 1. The Q-Q plots in S2A and S2B Fig show that the admixture mapping test statistics are approximately normally distributed except at the tails. The test statistics are least normally distributed for Asian Americans, which exhibits the most imbalance between cases and controls. The strongest peak of association observed was identified in a single region at chromosome 8 from 207,207–314,620 (GRCh37) in Hispanics that corresponds to an increase in European ancestry in cases compared to controls (S13–S15 Tables and Fig 8). This is the only peak that reached genome-wide significance with a Bonferroni adjusted p-value of 3.36 × 10^−2^. The closest gene to this region is *ZNF596*, a zinc finger protein 9.8 kb downstream that is most highly expressed in the brain and cerebellum out of 20 different human tissues whose total RNA was sequenced[14].

## Discussion

The genetic contribution to MS susceptibility is very complex; most studies have focused on populations of Northern European descent, and to date, the involvement of genes within and outside the MHC region has been established. Admixed individuals are derived from distinct ancestral populations; global and local genetic ancestry estimates can be used to test for association between the genome, a genetic locus or specific allele and a phenotype of interest[12,15,16]. This is one of the first studies to examine the relationship between genetic ancestry, HLA and non-HLA alleles and MS in three admixed populations: African Americans, Hispanics, and Asian Americans.

Within the MHC, we were first able to replicate the association of some previously established HLA risk alleles with MS[11]; HLA-DRB1*15:*01* was the most significant finding across all three admixed populations[5]. Here, the odds ratios (ORs) for HLA-DRB1*15:*01* observed in admixed populations (1.88–2.45) were slightly lower than described in previous reports for White, non-Hispanic individuals (2.92)[11], but the direction of effect is consistent. In African Americans, we further replicated the association and direction of effect of HLA alleles previously established in the White, non-Hispanic population: HLA-DRB1*03:*01*, HLA-A*02:*01*, HLA-DRB1*14:*01*, and HLA-B*38:*01* at nominal level significance (p < 0.05)[11]. Additionally, we replicated the African HLA risk allele HLA-DRB1*15:*03* in African Americans[9]. A similar study by Isobe, *et al*. also replicated the association of HLA alleles HLA-DRB1*15:*01*, HLA-DRB1*03:*01*, HLA-DRB1*15:*03*, and HLA*02:*01*. Although HLA-DRB1*14:*01* was not found by Isobe to be significantly associated (P-value = 0.070), its protective effect is consistent with what is observed in this study. In summary, we detected association for 5 of the 6 established HLA MS alleles expected to be replicated under power calculations, and this supports the hypothesis that the MS genetic risk in African Americans partially overlaps with that of Europeans[17]. In both Hispanics and Asian Americans, HLA-DRB1*15:*01* is the only established HLA risk allele in White, non-Hispanics that was replicated[11], which suggests a smaller overlap in MS genetic risk between Hispanics and Asian Americans with that of Europeans.

At a nominal level of significance (p < 0.05), analysis of the HLA alleles identified five candidate risk alleles and four candidate protective alleles for African Americans, nine candidate risk alleles and four candidate protective alleles for Hispanics, and two candidate risk alleles and one candidate protective allele for Asian Americans. All directions of effect (risk or protective) of candidate MS HLA alleles are the same if found in more than one admixed population. In total, four of the nine protective HLA alleles novel in this study for MS belong to class I genes and five are class II *DRB1* alleles. It is plausible that the lower prevalence of MS in some admixed populations could be partially explained by the effects of protective alleles.

Of the significantly associated HLA haplotypes and alleles reported by Mack, *et al*. in Europeans, three were nominally associated with MS in at least one admixed population in this study[18]. In particular, the HLA-DRB1*03:*01* and HLA-A*02:*01* alleles in African Americans exhibited similar ORs and direction of effect (Table 5). However, the HLA-C*03:*04* allele in Asian Americans conferred risk (OR = 1.69) instead of a protective effect (Table 7). It is plausible that this disagreement is because an overwhelming majority (95.7%) of HLA-C*0304 alleles in Asian Americans are of East Asian origin in this study, while the investigation by Mack, *et al*. was in European Americans (Fig 5C, S23 Table and Table 7). The exon differences observed between European and African HLA-DRB1*15:*01* suggests that future high-resolution HLA analysis could further explain the differences in risk and protective effects that is due to ancestry.

The entire MHC region spanning 29,570,005–33,377,701 (GRCh37) had a higher proportion of European ancestry in MS cases compared to controls for both African American and Hispanic populations. In Asian Americans, the MHC region had a higher proportion of East Asian ancestry in cases compared to controls. Interestingly, the local MHC ancestry associations observed in the current study for African Americans and Hispanics contrasted with global ancestry—African American and Hispanic cases demonstrated less European ancestry compared to controls when the whole genome was taken into consideration, and Asian American cases demonstrated more European ancestry compared to controls. To investigate these associations further, we characterized the admixture proportions of MS-associated HLA alleles. Fig 5 (S23 Table) shows that a majority of HLA alleles, including HLA-DRB1*15:*01*, were inferred to exist in multiple ancestries and could thus be considered cosmopolitan. African American cases were not significantly European at the class II region compared to controls likely due to the contribution of the common African allele HLA-DRB1*15:*03*. In Asian Americans, HLA-DRB1*15:*01* and HLA-C*03:*01* conferred risk of MS and accounted for 68.6% of HLA alleles associated with MS. Together, these two alleles had an average of 94.7% East Asian ancestry which helps explain why cases tended to have a higher proportion of East Asian ancestry compared to controls within the MHC region.

We find it noteworthy that the European HLA-DRB1*15:*01* allele confers three times the odds of MS compared to the African HLA-DRB1*15:*01* allele in the African Americans we studied. A similar effect has been observed for European HLA-B*07:*02* and HLA-A*03:01. Together these findings provide evidence that in some genetic regions, the European haplotype could confer more risk of MS than haplotypes derived from other ancestries. In these cases, it is plausible that disease-causing genetic variants can come from only one ancestral population. However, it must be noted that this has not been found to be true for all admixed MS-associated alleles we examined (S1 Table), and that for alleles such as the African MS risk allele HLA-DRB1*15:*03*, the African haplotype confers more risk than the European haplotype. These findings together further highlight the complex genetic ancestry of MS-associated alleles in admixed populations.

Given that HLA-DRB1*15:*01* is in very strong linkage disequilibrium with HLA-DQB1*06:*02* in Europeans, we investigated whether the increased risk of MS in African Americans conferred by European HLA-DRB1*15:*01* could possibly be due to HLA-DQB1*06:*02*, despite the limitation that *HLA-DQB1* did not pass our imputation quality cutoff (average R^2^ = 0.53 across all *DQB1* alleles). As expected, 99.5% of HLA-DRB1*15:*01* haplotypes that include HLA-DQB1*06:*02* in African Americans are of European ancestry. Counts of all observed DRB1*15:*01-DQB1* haplotypes are shown in S16 and S17 Tables. S1 Table shows that HLA-DQB1*06:*02* was not associated with MS in African Americans (OR = 1.14, 95% CI: 0.68–1.92, p = 0.72). Further, S3 Table shows that comparison of European HLA-DQB1*06:*02* alleles with African HLA-DQB1*06:*02* alleles, in the absence of HLA-DRB1*15:*01*, did not demonstrate evidence for a significant association (OR = 0.48, 95% CI: 0.23–1.00, p = 0.07); the direction of effect is, in fact, protective. Results from the current study are consistent with a previous report showing the association of MS with the HLA-DRB1*15:01-DQB1*06:*02* haplotype is due to the *DRB1* locus independent of DQB1*06:*02*[19].

A comparison of the most commonly imputed SNP and AA subsequences between European and African HLA-DRB1*15:*01* alleles revealed mismatches at exons 1, 3, and 5. Each of these exons help encode the DR beta 1 heterodimer, with exon 1 encoding the leader peptide and exon 5 encoding the cytoplasmic tail of the membrane protein. Exon 3, together with exon 2, encode the two extracellular domains[20]. Further investigation into whether genetic variation in these exons have functional consequences for peptide presentation in the context of MS is warranted. Our case study of HLA-DRB1*15:*01* illustrates how admixture mapping can be broadly applied to better characterize risk alleles in admixed populations.

Consistent with previous attempts to replicate the association of non-HLA genetic risk variants, we also failed to replicate association of most non-HLA genetic risk variants across all three admixed populations, except for rs405343 and rs6670198 in Asian Americans, which exhibit the same direction of effect as in whites[8,9,21]. Without correction for multiple testing with significance established at p < 0.05, we replicated the association of 13 SNPs in African Americans, 21 SNPs in Hispanics, and 28 SNPs in Asian Americans (S10–S12 Tables). For African Americans and Hispanics, we replicated less associations than is expected under power calculations. For Asian Americans, more associations were replicated than is expected. The majority of non-HLA MS risk variants identified so far appears to be cosmopolitan and their observed ancestry proportions are reflective of global admixture proportions (Fig 7, S24 Table). European global ancestry and European local ancestry at the non-HLA genetic risk loci was not correlated with the unweighted genetic risk score comprised of the non-HLA variants (S1 Fig, S25 Table). Although our investigation showed that the majority of non-HLA MS genetic risk variants reported for the White, non-Hispanic population do not demonstrate strong associations with MS in African Americans, Asian Americans, and Hispanics, our study is under-powered to detect most associations. Besides lacking power due to small sample and effect sizes, there are multiple other explanations for why we may fail to replicate many associations of the non-HLA genetic risk variants with MS[9]. One explanation is that differences in minor allele frequencies reduced the power to detect associations in admixed populations. Another explanation is that the smaller haplotype blocks of African Americans and Hispanics may have caused many non-HLA genetic risk variants to fail tagging the putative causative variant of MS. Lastly, the absence of replication could simply be due to genetic heterogeneity across populations, which further justifies the need for GWAS in non-White populations.

A genome-wide search for European ancestry differences between MS cases and controls in all three admixed populations resulted in one region of chromosome 8 from 207,207 to 314,620 (GRCh37) in Hispanics only. The closest gene to this region is *ZNF596*, a zinc finger protein 9.8 kb away that is highly expressed in the brain and cerebellum. Lesions in brain tissue as well as brain atrophy are pathological hallmarks of MS[22], and available data suggest Hispanics may have a more severe disease course than White, non-Hispanic individuals[23]; however, these findings await replication. Further investigation of this region in a larger independent dataset and full interrogation of nearby genes and determining whether *ZNF596* could be involved in MS pathogenesis from a functional perspective are warranted.

Some important strengths of this study included comprehensive analyses of a large, well-characterized dataset comprised of 12,384 admixed MS cases and controls with high quality genetic data, the application of rigorous quality control procedures, genetic imputation methods for both SNP and HLA loci, probabilistic graphical modeling for local admixture estimation across the genome, and non-parametric statistical testing to identify local admixture differences between cases and controls that accounts for global differences. In the current study, the combined analysis of SNP and HLA genotypes in African Americans revealed for the first time, strong evidence that the European HLA-DRB1*15:*01* allele confers three times the MS risk compared to the African HLA-DRB1*15:*01* allele. This finding indicates increased risk attributed to the European 15:01 allele could be due to functional differences within *DRB1* itself, or possibly due to variant(s) present on the European HLA-DRB1*15:*01* haplotype that are not found on the African haplotype.

Some limitations must also be acknowledged. The diagnosis of MS cases in this large dataset occurred over a twenty-five year period and in different clinical settings; both prevalent and incident cases were included. Although all cases fulfilled established diagnostic criteria, is not known whether local genetic ancestral proportions (of particular importance in the current study) would be expected to change for cases diagnosed at different time points; larger investigations would be needed. We performed MDS analysis of genotype data to broadly categorize samples as African Americans, Asian Americans, or Hispanics for case-control analysis; careful matching on self-reported race/ethnicity was not possible for all individuals. MDS components were therefore used in each analysis to control for potential confounding; however, it is possible that population stratification could still contribute to some of our findings. The Asian MS case sample utilized in the current study was small compared to the other groups, reflecting the low prevalence of disease in this population, which reduced power to detect to modest effects.

In conclusion, results from the current study reveal a complex picture of genetic ancestry for MS-associated alleles in African Americans, Asian Americans, and Hispanics. Our study shows that the higher prevalence of MS in populations of northern European ancestry cannot simply be explained by the European ancestral origin of genetic risk factors. Rather, any difference in prevalence due to genetics might be partially explained by a combination of European risk alleles exerting greater risk (i.e. HLA-DRB1*15:*01*) compared to non-European risk alleles, or the presence of protective alleles in individuals of non-European ancestry. However, this does not rule out the possibility that observed prevalence differences could result from the influence of environmental risk factors or socioeconomic status, including differences in access to neurologists and diagnostic protocols using MRI, that may be population-specific.

## Materials and methods

### Ethics statement

Institutional Review Board approval was obtained for this study by the UC Berkeley Committee on Protection of Human Subjects (CPHS) (2010-03-928); PROTOCOL TITLE: Genetic and non genetic risk factors for MS; UC San Francisco Human Research Protection Program Institutional Review Board (IRB) (10–05039); PROTOCOL TITLE: Environmental and genetic risk factors for pediatric multiple sclerosis; Kaiser Permanente Southern California IRB (5962); PROTOCOL TITLE: MS Sunshine Study; and Kaiser Permanente Northern California IRB (CN-03CScha-05-H); PROTOCOL TITLE: Genetic and Non-Genetic Predictors of Risk for Multiple Sclerosis. Written informed consent was obtained for all study participants at these sites.

### Sample collection and genotyping

Genotype data from a total of 21,647 subjects were collected from the Northern and Southern California Kaiser Permanente memberships, the U.S. Pediatric MS Network, the Genetic Epidemiology Research on Aging (GERA) cohort, and International Multiple Sclerosis Genetics Consortium (IMSGC). Table 9 shows the starting number of MS cases and controls by dataset. All cases met the diagnostic criteria for MS[24,25]. Subjects from Northern California Kaiser Permanente and U.S. Pediatric MS Network were genotyped on the Illumina Human660W-Quad BeadChip, Infinium Human OmniExpress BeadChip, and Infinium Human OmniExpress Exome BeadChip. Subjects from Southern California Kaiser Permanente were genotyped on OmniExpress platforms. The 1,265 African American subjects from IMSGC were genotyped using the Illumina Immunochip and combined with other African Americans to study the ancestry of the MHC region[8]. Note that IMSGC subjects were not genotyped genome-wide and were thus excluded from the genome-wide studies in this paper.

**Table 9:** Dataset sources for admixed populations.

Source	Case (n)	Control (n)
Northern California Kaiser Permanente	1,069	637
Southern California Kaiser Permanente	645	636
U.S. Pediatric MS Network	792	413
IMSGC ImmunoChip	803	462
Genetic Epidemiology Research on Aging	0	16,168
Total	3,320	18,327

Starting number of cases and controls for each dataset source. n = number of individuals.

Genotyping details for the GERA cohort are described elsewhere[26]. All genetic coordinates were converted to NCBI Build 37 before analysis. BEAGLE was used to obtain phased data for African Americans, Asian Americans, and Hispanics independently, using GRCh37 genetic map positions in centimorgans converted from GRCh37 genetic coordinates by BEAGLE utility software. Genetic map positions capture genetic linkage information and is used by RFMix for defining windows for local ancestry assignment. The reference panel used for phasing was constructed from selecting individuals from 1000 Genomes with ancestries present in each admixed population[27,28]. The ancestries represented in our dataset were European (present in all groups), African (present in African Americans and Hispanics), East Asian (present in Asian Americans), and Native American (present in Hispanics).

### Imputation

Genome-wide imputation of the dataset against the entire 1000 Genomes phase 3 reference panel was carried out using IMPUTE2[27,29]. For HLA imputation, SNP2HLA was used to perform 2-field imputation of alleles for *HLA-A*, *HLA-B*, *HLA-C*, *DRB1*, and *DQB1* using an admixed reference panel from the 1000 Genomes Project, comprised of 165 Native Americans, 155 Africans, 251 East Asians, and 303 Europeans[27,30,31]. The reference panel was tailored to contain ancestries represented by the target population to enhance imputation accuracy, and HLA alleles in each admixed population were imputed independently as previously described[32].

### Quality control

SNPs were filtered for minor allele frequency (> 0.01) and missingness on SNPs and samples (> 0.10) before and after imputation with IMPUTE2. Genotype probabilities from IMPUTE2 were converted to hard genotype calls using > 0.6 as the threshold, and SNPs were filtered for info score > 0.30. Additionally, A/T and C/G SNPs were discarded prior to local ancestry inference to avoid strand ambiguity. Related individuals ($\hat{\pi }>0.25$) were removed from further analysis, resulting in a total of 20,588 samples. For HLA imputation using SNP2HLA, we removed alleles with R^2^ scores less than 0.80 and with allele frequencies below 0.005 from further analysis, filtering out 40, 66, and 63 HLA alleles to result in 70, 47, and 77 HLA alleles for African Americans, Asian Americans, and Hispanics, respectively. All quality control (QC) steps were performed using the PLINK software and R v3.3.1 (www.r-project.org)[33].

### Analysis of population structure

Population structure was assessed using MDS and fastSTRUCTURE prior to genotype imputation in order to divide the samples into African American, Asian American, or Hispanic groups for further analysis[34]. MDS components captured ancestry to identify individuals likely to be African American, Asian American, or Hispanic, using reference populations from the Human Genome Diversity Project (HGDP)[35]. Subjects that cluster with the European reference samples were identified as White, non-Hispanic and subsequently removed. Then, fastSTRUCTURE was used for each group to estimate global admixture proportions for individuals using independent SNPs and a HGDP reference panel tailored to the target population, with default parameters. A cutoff of at least 5% Native American global ancestry for Hispanics was imposed to further remove White, non-Hispanic individuals who were removed based on MDS. The 1,163 candidate Hispanic individuals who did not meet this requirement had an average 0.7% Native American ancestry and 96% European ancestry.

### Local ancestry inference

We inferred local ancestry genome-wide separately for African Americans, Asian Americans, and Hispanics using RFMix software analysis of imputed and phased genotype data, and a reference panel from the 1000 Genomes Project tailored to the target population[27,36]. The 1000 Genomes reference panel was selected over the HDGP reference panel as the appropriate reference because it has the required high genotype density for local ancestry inference. RFMix was run on recommended input parameters of 5 minimum number of reference haplotypes per tree node and 3 EM iterations. The number of generations of admixture used as input parameters for RFMix were 5, 6, and 11 for Asian Americans, African Americans, and Hispanics, respectively, according to previous estimates for populations in the United States [37].

### Statistical analysis

Association testing between case status and genetic ancestry was performed using the nonparametric test statistic proposed by Montana and Pritchard for admixture mapping[38].

$$T\left(l,\;k\right)=\frac{\left({\bar{z}}_{l,\;d}\left(k\right)-\;{\bar{z}}_{l,\;c}\left(k\right)\right)-\left({\bar{q}}_{d}\left(k\right)-\;{\bar{q}}_{c}\left(k\right)\right)}{SD\left({\bar{z}}_{l,\;d}\left(k\right)-\;{\bar{z}}_{l,\;c}\left(k\right)\right)}$$ (1)

Briefly, the term ${\overset{-}{z}}_{l,\;d}(k)$ represents the average local ancestry of cases at locus *l* for ancestry *k* and ${\overset{-}{z}}_{l,\;c}(k)$ is similarly defined for controls. The term ${\overset{-}{q}}_{d}(k)$ represents the genome-wide average of ancestry *k* among cases and ${\overset{-}{q}}_{c}(k)$ is defined similarly for controls. Genome-wide ancestry estimates for this statistic are taken from local ancestry estimates from RFMix. This test statistic can be used to test for ancestry association at a single locus or at a region. Under the null, the test statistic follows the normal distribution and a P value can be obtained through a z-test. The variance $Var({\overset{-}{z}}_{l,\;d}(k)-\;{\overset{-}{z}}_{l,\;c}(k))$ of the test statistic at a given locus was empirically estimated as the sum of variance of average ancestry among cases and controls. The standard deviation follows as the square root of the variance. This estimation corresponds to estimating the standard deviation of the average treatment effect, with disease status as treatment and ancestry as outcome[39]. All terms of the test statistic were estimated from local ancestry estimates from RFMix. Complete details are described elsewhere[38].

Multivariate logistic regression was applied to evaluate the association of genetic variants with MS, using an additive model and adjusting for the first three MDS components to control for population stratification[8,9]. ORs were used to characterize effect sizes of MS risk alleles. The Wilcoxon test was used to evaluate significance of global admixture proportion differences between cases and controls. All analyses were performed using PLINK and R v3.3.1 (www.r-project.org)[33].

Multiple hypothesis testing was addressed with Bonferroni correction. Bonferroni correction was used to establish significance for the study of non-HLA alleles, and adjusted p-values were provided for all multiple testing scenarios except when the number of tests is ten or less. For genome-wide association studies, a significance level of α = 0.05 with 15,282 tests results in a genome-wide significance level of 3.27 × 10^−6^. Bonferroni correction was applied independently for the studies of African Americans, Hispanics, and Asian Americans.

Since local ancestry assignments span multiple loci, we reduced the burden of multiple hypothesis testing for ancestry association across the genome by only testing one locus per window defined by RFMix for inferring local ancestry, resulting in a total of 15,282 tests genome-wide. Complete details of how RFMix defines windows for local ancestry inference is described elsewhere[36].

### Power calculations

Power calculations are performed with the Genetic Association Study Power Calculator (http://csg.sph.umich.edu/abecasis/cats/gas_power_calculator/), which implements calculations from Skol *et al*.[40]. We assume an additive disease model, a MS prevalence of 0.1% in the United States, significance level of 5%, and disease allele frequency of 10%[41]. For HLA alleles, we assume a relative risk of 2, and a relative risk of 1.2 for non-HLA alleles.

### Comparison of SNP and amino acid subsequences

SNPs and AAs imputed by SNP2HLA for European and African HLA-DRB1*15:*01* alleles in African Americans were aligned to the UCSC Genome Browser GRCh38 RefSeq Genes track, and the European subsequences were compared to the African subsequences. Note that “subsequence” refers to only the imputed SNPs and AAs, and not to contiguous DNA or AA sequence.

## Supporting information

S1 Fig Unweighted genetic risk score versus European ancestry. Plot of unweighted genetic risk score of the MS genetic risk variants passing QC versus European ancestry globally and locally at the corresponding MS genetic risk loci. All ancestries were estimated with RFMix. Panels (A), (C), and (E) shows the relationship between risk score and percentage European global ancestry for African Americans, Hispanics, and Asian Americans respectively. Panels (B), (D), and (F) shows the relationship between risk score and percentage European local ancestry calculated at the MS genetic risk loci for African Americans, Hispanics, and Asian Americans. There was little correlation (R^2^ < 0.30; P-value > 0.05) between genetic risk score and European ancestry.
(TIFF)

S2 Fig Q-Q plot of admixture mapping test statistic. Q-Q plot of admixture mapping test statistic for (A) African Americans, (B) Hispanics, and (C) Asian Americans. The line y = x represents the theoretical Q-Q plot if the test statistics are perfectly normally distributed.
(TIFF)

S1 Table Odds ratio of MS for European allele versus African allele. Odds ratio (OR) of European HLA allele to African HLA allele as determined from logistic regression for African American MS-associated alleles, adjusting for first 3 MDS components. OR are shown with 95% confidence interval and corresponding p-values. HLA alleles with sample size less than 50 or with predominant ancestry greater than 90% are excluded from the analysis. Furthermore, alleles not inferred to be completely European or African are excluded, and only alleles from individuals with one copy are included.
(PDF)

S2 Table HLA-DRB1*15:*01* haplotypes in African Americans. Two-by-two table of counts of DRB1*15:*01–DQB1* haplotypes where DRB1*15:*01* is European and the identity of the *DQB1* allele on the haplotype is summarized. All HLA alleles had allele frequency greater than 0.005, and only DRB1*15:*01* alleles that were completely European were considered. DQB1*X denotes any *DQB1* allele that is not DQB1*06:*02*, and that there is no restriction on the ancestry of the *DQB1* allele. Note: *DQB1* alleles did not pass imputation quality cutoff of r^2^ = 0.80 (see text for details).
(PDF)

S3 Table European and African HLA-DQB1*06:*02* haplotypes in African Americans. Two-by-two table of counts of DRB1*X–DQB1*06:*02* haplotypes where DRB1*X denotes any allele other than DRB1*15:*01*. All HLA alleles had allele frequency greater than 0.005, and only *DQB1* alleles that were either completely European or African are considered. There is no restriction on the ancestry of the *DRB1* allele. Note: *DQB1* alleles did not pass imputation quality cutoff of r^2^ = 0.80 (see text for details).
(PDF)

S4 Table Imputed European HLA-DRB1*15:*01* SNP subsequences in African Americans. Imputed SNPs for all European HLA-DRB1*15:*01* alleles in African Americans. SNPs are listed left to right in order of increasing genetic coordinates. Note that imputed SNPs are not contiguous and imputation was performed by SNP2HLA.
(PDF)

S5 Table Imputed African HLA-DRB1*15:*01* SNP subsequences in African Americans. Imputed SNPs for all African HLA-DRB1*15:*01* alleles in African Americans. SNPs are listed left to right in order of increasing genetic coordinates. Note that imputed SNPs are not contiguous and imputation was performed by SNP2HLA.
(PDF)

S6 Table Imputed European HLA-DRB1*15:*01* amino acid subsequences in African Americans. Imputed amino acids (AA) for all European HLA-DRB1*15:*01* alleles in African Americans. AAs are listed left to right in order of increasing genetic coordinates. Note that imputed AAs are not contiguous and imputation was performed by SNP2HLA.
(PDF)

S7 Table Imputed African HLA-DRB1*15:*01* amino acid subsequences in African Americans. Imputed amino acids (AA) for all African HLA-DRB1*15:*01* alleles in African Americans. AAs are listed left to right in order of increasing genetic coordinates. Note that imputed AAs are not contiguous and imputation was performed by SNP2HLA.
(PDF)

S8 Table Genetic coordinates of imputed HLA-DRB1*15:*01* SNPs. Genetic coordinates in GRCh37 of SNPs imputed by SNP2HLA for HLA-DRB1*15:*01*.
(XLSX)

S9 Table Coordinates of imputed HLA-DRB1*15:*01* amino acids. Genetic coordinates in GRCh37 and amino acid positions of amino acids (AA) imputed by SNP2HLA for HLA-DRB1*15:*01*. Note that in SNP2HLA, amino acid position 1 starts from exon 2.
(XLSX)

S10 Table Association signals of non-HLA MS risk variants in African Americans. Association testing of the established MS genetic risk variants with MS in African Americans. Association is evaluated for local European ancestry at risk loci and separately for MS risk variants. Only risk variants that pass QC are tested. Abbreviations: OR = odds ratio; p value (RV) = p value from testing of association between MS risk variant and MS; adj p value (RV) = Bonferroni-adjusted p value (RV); z score = admixture mapping test statistic; p value (EUR) = p value from testing of association between European ancestry and MS; adj p value (EUR) = Bonferroni-adjusted p value (EUR). Positions are in NCBI build-37 coordinates.
(XLSX)

S11 Table Association signals of non-HLA MS risk variants in Hispanics. Association testing of the established MS genetic risk variants with MS in Hispanics. Association is evaluated for local European ancestry at risk loci and separately for MS risk variants. Only risk variants that pass QC are tested. Abbreviations: OR = odds ratio; p value (RV) = p value from testing of association between MS risk variant and MS; adj p value (RV) = Bonferroni-adjusted p value (RV); z score = admixture mapping test statistic; p value (EUR) = p value from testing of association between European ancestry and MS; adj p value (EUR) = Bonferroni-adjusted p value (EUR). Positions are in NCBI build-37 coordinates.
(XLSX)

S12 Table Association signals of non-HLA MS risk variants in Asian Americans. Association testing of the established MS genetic risk variants with MS in Asian Americans. Association is evaluated for local European ancestry at risk loci and separately for MS risk variants. Only risk variants that pass QC are tested. Abbreviations: OR = odds ratio; p value (RV) = p value from testing of association between MS risk variant and MS; adj p value (RV) = Bonferroni-adjusted p value (RV); z score = admixture mapping test statistic; p value (EUR) = p value from testing of association between European ancestry and MS; adj p value (EUR) = Bonferroni-adjusted p value (EUR). Positions are in NCBI build-37 coordinates.
(XLSX)

S13 Table Top association signals of genome-wide admixture mapping in African Americans. Top association signals from genome-wide scan of association between local European ancestry and MS. The z score and p value from admixture mapping are shown. adj p value = Bonferroni-adjusted p value. Positions are in NCBI build 37 coordinates.
(XLSX)

S14 Table Top association signals of genome-wide admixture mapping in Hispanics. Top association signals from genome-wide scan of association between local European ancestry and MS. The z score and p value from admixture mapping are shown. adj p value = Bonferroni-adjusted p value. Positions are in NCBI build 37 coordinates.
(XLSX)

S15 Table Top association signals of genome-wide admixture mapping in Asian Americans. Top association signals from genome-wide scan of association between local European ancestry and MS. The z score and p value from admixture mapping are shown. adj p value = Bonferroni-adjusted p value. Positions are in NCBI build 37 coordinates.
(XLSX)

S16 Table European DRB1*15:01– DQB1*X haplotypes in African Americans. *HLA-DQB1* alleles that European HLA-DRB1*15:01 is linked to in African Americans. All HLA alleles have frequency equal to or greater than 0.005, and there is no constraint on the ancestry of *HLA-DQB1* alleles. *X* = wildcard for any *HLA-DQB1* allele.
(PDF)

S17 Table African DRB1*15:*01– DQB1* haplotypes in African Americans. *HLA-DQB1* alleles that African HLA-DRB1*15:*01* is linked to in African Americans. All HLA alleles have frequency equal to or greater than 0.005, and there is no constraint on the ancestry of *HLA-DQB1* alleles. *X* = wildcard for any *HLA-DQB1* allele.
(PDF)

S18 Table MS genome-wide genetic variant associations outside the major histocompatibility complex region. (PDF)

S19 Table MDS components of study subjects and HGDP reference samples. Supporting information for Fig 1.
(XLSX)

S20 Table Average global ancestry proportions by admixed population. Supporting information for Fig 2.
(XLSX)

S21 Table Change in European ancestry vs genome average in MHC region by admixed population. Supporting information for Fig 3.
(XLSX)

S22 Table P-values of MS-associated HLA alleles by admixed population. Supporting information for Fig 4.
(XLSX)

S23 Table Ancestry proportions of HLA alleles associated with MS by admixed population. Supporting information for Fig 5.
(XLSX)

S24 Table Ancestry proportions of non-HLA MS risk variants by admixed population. Supporting information for Fig 7.
(XLSX)

S25 Table MS risk score and European ancestry by admixed population. Supporting information for S1 Fig.
(XLSX)
