# Supplementary figures and images for: Admixture mapping reveals evidence of differential multiple sclerosis risk by genetic ancestry
Source: PLoS Genet. 2019 Jan 17;15(1):e1007808. doi: 10.1371/journal.pgen.1007808 (PMC6353231; doi:10.1371/journal.pgen.1007808)

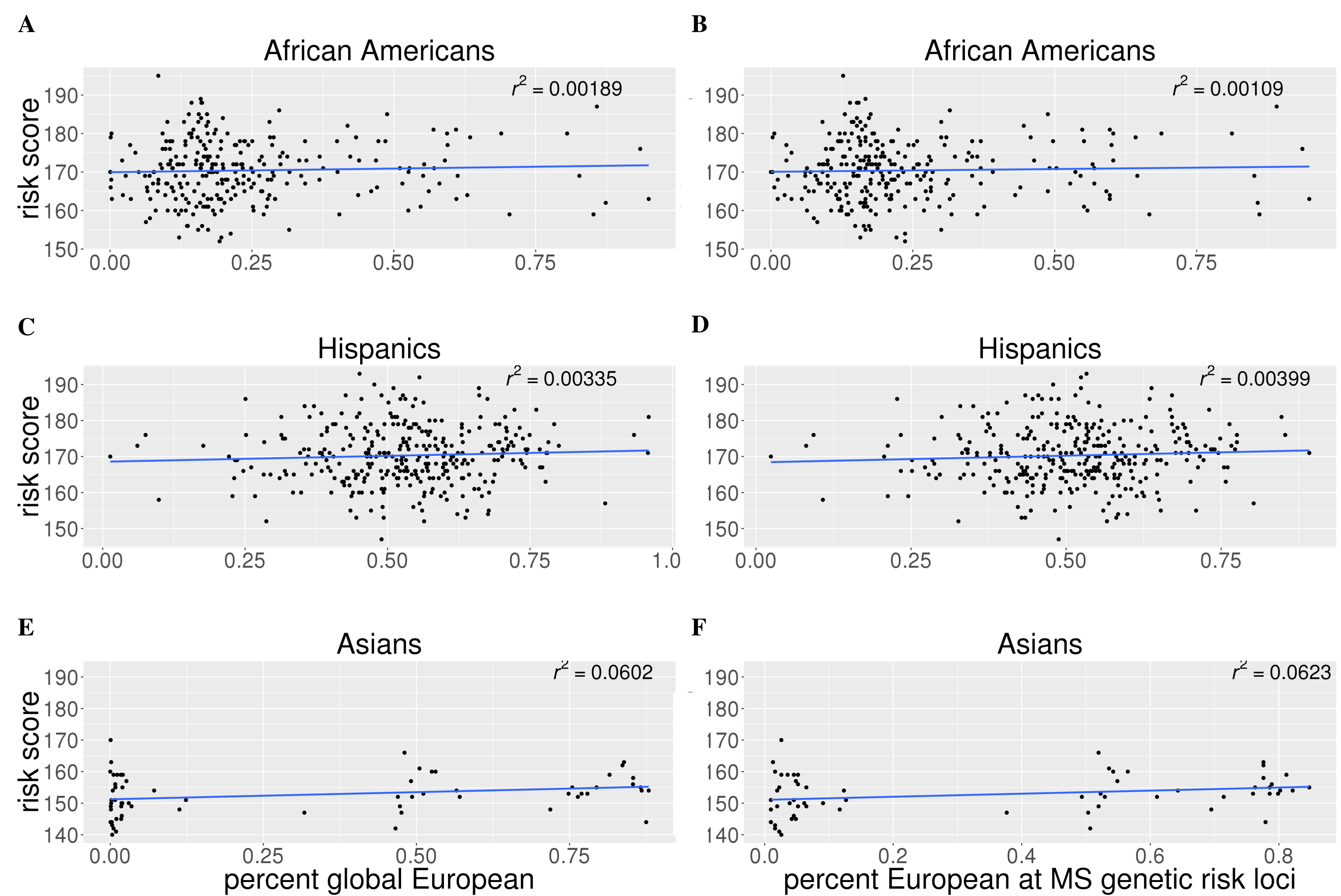

Supplement: S1 Fig — Plot of unweighted genetic risk score of the MS genetic risk variants passing QC versus European ancestry globally and locally at the corresponding MS genetic risk loci. All ancestries were estimated with RFMix. Panels (A), (C), and (E) shows the relationship between risk score and percentage European global ancestry for African Americans, Hispanics, and Asian Americans respectively. Panels (B), (D), and (F) shows the relationship between risk score and percentage European local ancestry calculated at the MS genetic risk loci for African Americans, Hispanics, and Asian Americans. There was little correlation (R2 < 0.30; P-value > 0.05) between genetic risk score and European ancestry. (TIFF) [file pgen.1007808.s001.tiff]

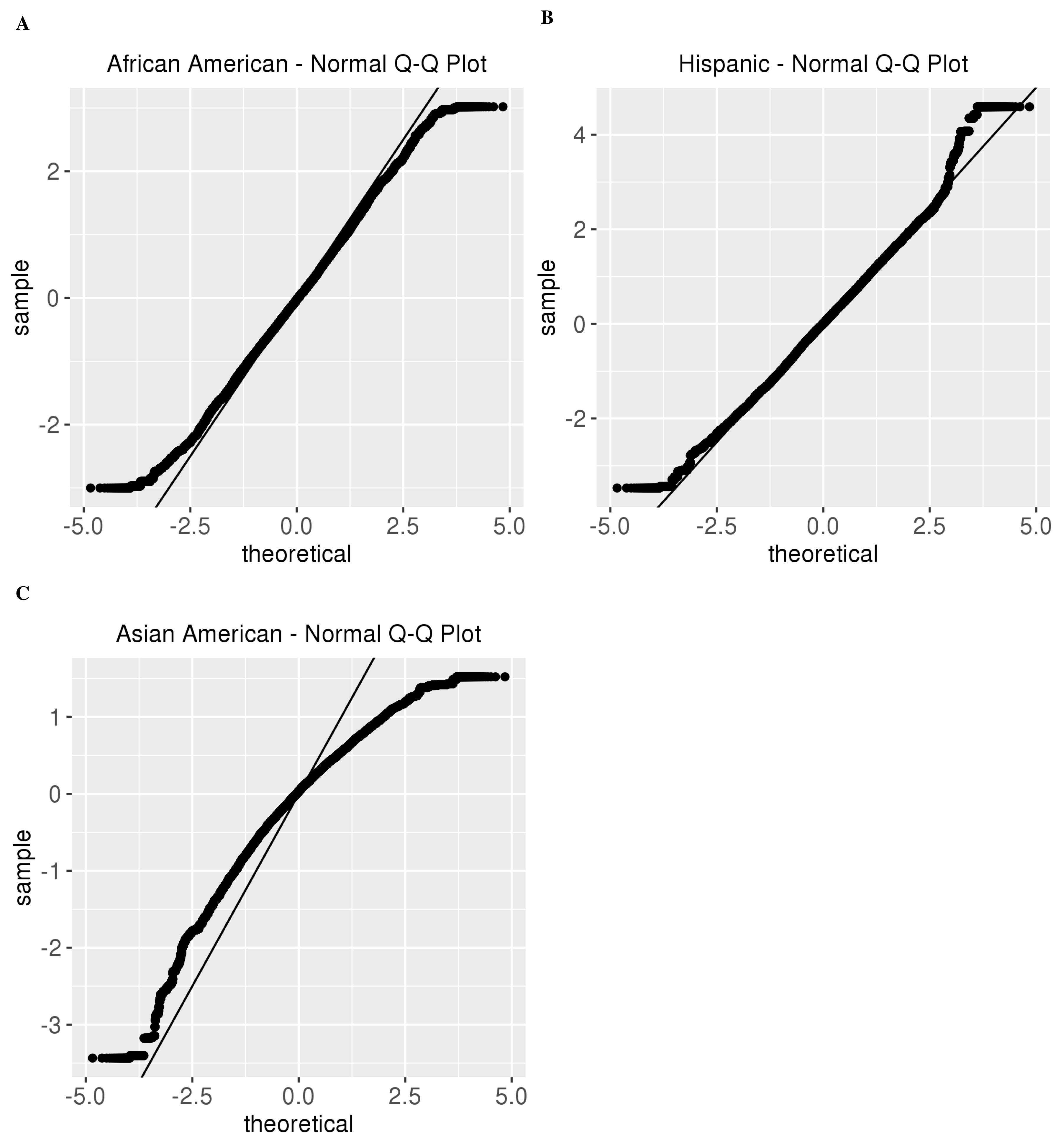

Supplement: S2 Fig — Q-Q plot of admixture mapping test statistic for (A) African Americans, (B) Hispanics, and (C) Asian Americans. The line y = x represents the theoretical Q-Q plot if the test statistics are perfectly normally distributed. (TIFF) [file pgen.1007808.s002.tiff]
