# Supplementary material for: Admixture mapping reveals evidence of differential multiple sclerosis risk by genetic ancestry
Source: PLoS Genet. 2019 Jan 17;15(1):e1007808. doi: 10.1371/journal.pgen.1007808 (PMC6353231; doi:10.1371/journal.pgen.1007808)
Supplement: S1 Table — Odds ratio (OR) of European HLA allele to African HLA allele as determined from logistic regression for African American MS-associated alleles, adjusting for first 3 MDS components. OR are shown with 95% confidence interval and corresponding p-values. HLA alleles with sample size less than 50 or with predominant ancestry greater than 90% are excluded from the analysis. Furthermore, alleles not inferred to be completely European or African are excluded, and only alleles from individuals with one copy are included. (PDF) [file pgen.1007808.s003.pdf]

S1 Table. Odds Ratio of MS for European Allele versus African Allele

HLA Allele	OR	P value
DRB1*15:01	3.01 (1.90 – 4.75)	2.49E-6
DRB1*03:01	0.64 (0.43 – 0.96)	3.03E-2
A*02:01	0.91 (0.63 – 1.31)	5.94E-1
DRB1*14:01	0.40 (0.09 – 1.74)	2.24E-1
B*07:02	1.66 (1.12 – 2.47)	1.18E-2
A*03:01	1.54 (1.04 – 2.29)	2.97E-2
C*08:02	0.66 (0.29 – 1.54)	3.37E-1
C*04:01	1.07 (0.72 – 1.59)	7.41E-1
C*07:02	1.12 (0.75 – 1.67)	5.82E-1

Odds ratio (OR) of European HLA allele to African HLA allele as determined from logistic regression for African American MS-associated alleles, adjusting for first 3 MDS components. OR are shown with 95% confidence interval and corresponding p-values. HLA alleles with sample size less than 50 or with predominant ancestry greater than 90% are excluded from the analysis. Furthermore, alleles not inferred to be completely European or African are excluded, and only alleles from individuals with one copy are included.
