# Supplementary material for: Admixture mapping reveals evidence of differential multiple sclerosis risk by genetic ancestry
Source: PLoS Genet. 2019 Jan 17;15(1):e1007808. doi: 10.1371/journal.pgen.1007808 (PMC6353231; doi:10.1371/journal.pgen.1007808)
Supplement: S2 Table — Two-by-two table of counts of DRB1*15:01–DQB1 haplotypes where DRB1*15:01 is European and the identity of the DQB1 allele on the haplotype is summarized. All HLA alleles had allele frequency greater than 0.005, and only DRB1*15:01 alleles that were completely European were considered. DQB1*X denotes any DQB1 allele that is not DQB1*06:02, and that there is no restriction on the ancestry of the DQB1 allele. Note: DQB1 alleles did not pass imputation quality cutoff of r2 = 0.80 (see text for details). (PDF) [file pgen.1007808.s004.pdf]

S2 Table. *HLA-DRB1*15:01* haplotypes in African Americans

DRB1–DQB1 Haplotype	Case (n)	Control (n)	
EUR *DRB1*15:01 – DQB1*06:02*	99	142	241
EUR *DRB1*15:01–DQB1*X*	30	49	79
	129	191	320

Two-by-two table of counts of *DRB1*15:01–DQB1* haplotypes where *DRB1*15:01* is European and the identity of the *DQB1* allele on the haplotype is summarized. All HLA alleles had allele frequency greater than 0.005, and only *DRB1*15:01* alleles that were completely European were considered. *DQB1*X* denotes any *DQB1* allele that is not *DQB1*06:02*, and that there is no restriction on the ancestry of the *DQB1* allele. Note: *DQB1* alleles did not pass imputation quality cutoff of $r^2 = 0.80$ (see text for details).
