# Supplementary material for: Admixture mapping reveals evidence of differential multiple sclerosis risk by genetic ancestry
Source: PLoS Genet. 2019 Jan 17;15(1):e1007808. doi: 10.1371/journal.pgen.1007808 (PMC6353231; doi:10.1371/journal.pgen.1007808)
Supplement: S3 Table — Two-by-two table of counts of DRB1*X–DQB1*06:02 haplotypes where DRB1*X denotes any allele other than DRB1*15:01. All HLA alleles had allele frequency greater than 0.005, and only DQB1 alleles that were either completely European or African are considered. There is no restriction on the ancestry of the DRB1 allele. Note: DQB1 alleles did not pass imputation quality cutoff of r2 = 0.80 (see text for details). (PDF) [file pgen.1007808.s005.pdf]

S3 Table. European and African *HLA-DQB1*06:02* haplotypes in African Americans

DRB1–DQB1 Haplotype	Case (n)	Control (n)	
*DRB1*X* –EUR *DQB1*06:02*	10	38	48
*DRB1*X*–AFR *DQB1*06:02*	137	252	389
	147	290	437

Two-by-two table of counts of *DRB1*X–DQB1*06:02* haplotypes where *DRB1*X* denotes any allele other than *DRB1*15:01*. All HLA alleles had allele frequency greater than 0.005, and only *DQB1* alleles that were either completely European or African are considered. There is no restriction on the ancestry of the *DRB1* allele. Note: *DQB1* alleles did not pass imputation quality cutoff of $r^2 = 0.80$ (see text for details).
