# Supplementary material for: Admixture mapping reveals evidence of differential multiple sclerosis risk by genetic ancestry
Source: PLoS Genet. 2019 Jan 17;15(1):e1007808. doi: 10.1371/journal.pgen.1007808 (PMC6353231; doi:10.1371/journal.pgen.1007808)
Supplement: S4 Table — Imputed SNPs for all European HLA-DRB1*15:01 alleles in African Americans. SNPs are listed left to right in order of increasing genetic coordinates. Note that imputed SNPs are not contiguous and imputation was performed by SNP2HLA. (PDF) [file pgen.1007808.s006.pdf]

S4 Table. Imputed European *HLA-DRB1*15:01* SNP Subsequences in African Americans

SNP Subsequence	Counts
GTAGATTGCGCTCGCTTCGACGGGGGTATTTGGGTCAAG<br>TAGGCGCGCCTGCTTAAGGTCCAAACGACTAATAGCGAC<br>AGCCCCTCTGGCGCAGCGACGTGT	301
GTAGATCACATCCGCTCCGCCGGGGAGCCCAGGTTACAG<br>TAGGCGCGCCTGCTTAAGGTCCAAACGACTAATAGCGAC<br>AGCCCCTCTGGCGCAGCTCAACGC	9
GTAGATTGCGCTCGCTTCGACGGGGGTATTTGGGTCAAG<br>TAGGCGCGCCTGCTTAAGGTCCAAACGACTAATAGCGAC<br>AGCCCCTCTGGCGCAGCTCAACGC	6
GTAGATTGCGCTCGCTTCGACGGGGGTATTTGGGTCAAG<br>TAGGCGCGCCTGCTTAAGGTCCAAACGACTAATAGCGAC<br>AGCCCCTCGGAGATCACTCAACGC	4

Imputed SNPs for all European *HLA-DRB1*15:01* alleles in African Americans. SNPs are listed left to right in order of increasing genetic coordinates. Note that imputed SNPs are not contiguous and imputation was performed by SNP2HLA.
