# Supplementary material for: Admixture mapping reveals evidence of differential multiple sclerosis risk by genetic ancestry
Source: PLoS Genet. 2019 Jan 17;15(1):e1007808. doi: 10.1371/journal.pgen.1007808 (PMC6353231; doi:10.1371/journal.pgen.1007808)
Supplement: S5 Table — Imputed SNPs for all African HLA-DRB1*15:01 alleles in African Americans. SNPs are listed left to right in order of increasing genetic coordinates. Note that imputed SNPs are not contiguous and imputation was performed by SNP2HLA. (PDF) [file pgen.1007808.s007.pdf]

S5 Table. Imputed African *HLA-DRB1*15:01* SNP Subsequences in African Americans

SNP Subsequence	Counts
GTAGATTGCGCTCGCTTCGACGGGGGTATTTGGGTCAAGTAGGCGCGCCTGCTTAAGGTCCAAACGACTAATAGCGACAGCCCCTCTGGCGCAGCTCAACGC	107
CTAGATTGCGCCCGGTCTACCAGGGGTACTAGGGTCAAGTAGGCGCGCCTGCTTAAGGTCCAAACGACTAATAGCGACAGCCCCTCTGGCGCAGCTCAACGC	50
GTAGATTGCGCTCGCTTCGACGGGGGTATTTGGGTCAAGTAGGCGCGCCTGCTTAAGGTCCAAACGACTAATAGCGACAGCCCCTCTGGCGCAGCGACGTGT	12
GTAGATCACATCCGCTCCGCCGGGGAGCCCAGGTTACAGTAGGCGCGCCTGCTTAAGGTCCAAACGACTAATAGCGACAGCCCCTCTGGCGCAGCTCAACGC	7
CTAGATTGCGCCCGGTCTACCACGAGTACTTGGGTCAAGTAGGCGCGCCTGCTTAAGGTCCAAACGACTAATAGCGACAGCCCCTCTGGCGCAGCGACGTGT	2
CTAGATTGCGCCCGGTCTACCAGGGGTACTAGGGTCAAGTAGGCGCGCCTGCTTAAGGTCCAAACGACTAATAGCGACAGCCCCTCTGGCGCAGCGCCGTGT	1
GTAGATTGCGCTCGCTTCGACGGGGGTATTTGGGTCAAGTAGGCGCGCCTGCTTAAGGTCCAAACGACTAATAGCGACAGCCCCTCGGAGATCACTCAACGC	1

Imputed SNPs for all African *HLA-DRB1*15:01* alleles in African Americans. SNPs are listed left to right in order of increasing genetic coordinates. Note that imputed SNPs are not contiguous and imputation was performed by SNP2HLA.
