# Supplementary material for: Admixture mapping reveals evidence of differential multiple sclerosis risk by genetic ancestry
Source: PLoS Genet. 2019 Jan 17;15(1):e1007808. doi: 10.1371/journal.pgen.1007808 (PMC6353231; doi:10.1371/journal.pgen.1007808)
Supplement: S6 Table — Imputed amino acids (AA) for all European HLA-DRB1*15:01 alleles in African Americans. AAs are listed left to right in order of increasing genetic coordinates. Note that imputed AAs are not contiguous and imputation was performed by SNP2HLA. (PDF) [file pgen.1007808.s008.pdf]

Table S6. Imputed European *HLA-DRB1*15:01* Amino Acid Subsequences in African Americans

Amino Acid Subsequence	Counts
TQRTVRQMALSHSKQVVYTAAAQIYADFFVSNYFYDFRHER KPQWRSATLK	301
TQRMVRQVARSHAEHGVYTAAAQIYADFFVSNYFYDFRHER KPQWRAVALR	9
TQRTVRQMALSHSKQVVYTAAAQIYADFFVSNYFYDFRHER KPQWRAVALR	6
TQRTVRQMALSHSKQVVYTAAAQIYADFFVSNYFYDFRHER TSYERAVALR	4

Imputed amino acids (AA) for all European *HLA-DRB1*15:01* alleles in African Americans. AAs are listed left to right in order of increasing genetic coordinates. Note that imputed AAs are not contiguous and imputation was performed by SNP2HLA.
