# Supplementary material for: Admixture mapping reveals evidence of differential multiple sclerosis risk by genetic ancestry
Source: PLoS Genet. 2019 Jan 17;15(1):e1007808. doi: 10.1371/journal.pgen.1007808 (PMC6353231; doi:10.1371/journal.pgen.1007808)
Supplement: S7 Table — Imputed amino acids (AA) for all African HLA-DRB1*15:01 alleles in African Americans. AAs are listed left to right in order of increasing genetic coordinates. Note that imputed AAs are not contiguous and imputation was performed by SNP2HLA. (PDF) [file pgen.1007808.s009.pdf]

S7 Table. Imputed African *HLA-DRB1*15:01* Amino Acid Subsequences in African Americans

Amino Acid Subsequence	Counts
TQRTVRQMALSHSKQVVYTAAAQIYADFFVSNYFYDFRHER KPQWRAVALR	107
RQRTVRHVTRSHSKHVVYTAAAQIYADFFVSNYFYDFRHER KPQWRAVALR	50
TQRTVRQMALSHSKQVVYTAAAQIYADFFVSNYFYDFRHER KPQWRSATLK	12
TQRMVRQVARSHAEHGVYTAAAQIYADFFVSNYFYDFRHER KPQWRAVALR	7
RQRTVRHVTRSYSKQVVYTAAAQIYADFFVSNYFYDFRHER KPQWRSATLK	2
RQRTVRHVTRSHSKHVVYTAAAQIYADFFVSNYFYDFRHER KPQWRAATLK	1
TQRTVRQMALSHSKQVVYTAAAQIYADFFVSNYFYDFRHER TSYERAVALR	1

Imputed amino acids (AA) for all African *HLA-DRB1*15:01* alleles in African Americans. AAs are listed left to right in order of increasing genetic coordinates. Note that imputed AAs are not contiguous and imputation was performed by SNP2HLA.
