# Supplementary material for: Admixture mapping reveals evidence of differential multiple sclerosis risk by genetic ancestry
Source: PLoS Genet. 2019 Jan 17;15(1):e1007808. doi: 10.1371/journal.pgen.1007808 (PMC6353231; doi:10.1371/journal.pgen.1007808)
Supplement: S17 Table — HLA-DQB1 alleles that African HLA-DRB1*15:01 is linked to in African Americans. All HLA alleles have frequency equal to or greater than 0.005, and there is no constraint on the ancestry of HLA-DQB1 alleles. X = wildcard for any HLA-DQB1 allele. (PDF) [file pgen.1007808.s019.pdf]

Table S17. African *DRB1*15:01– DQB1* Haplotypes in African Americans

African *DRB1*15:01–DQB1*X*	Counts
*HLA-DQB1*02:01*	58
*HLA-DQB1*05:01*	55
*HLA-DQB1*05:02*	43
*HLA-DQB1*03:01*	14
*HLA-DQB1*03:02*	7
*HLA-DQB1*06:03*	2
*HLA-DQB1*06:02*	1

HLA-DQB1 alleles that African *HLA-DRB1*15:01* is linked to in African Americans. All HLA alleles have frequency equal to or greater than 0.005, and there is no constraint on the ancestry of *HLA-DQB1* alleles. *X* = wildcard for any *HLA-DQB1* allele.
