# Supplementary material for: Admixture mapping reveals evidence of differential multiple sclerosis risk by genetic ancestry
Source: PLoS Genet. 2019 Jan 17;15(1):e1007808. doi: 10.1371/journal.pgen.1007808 (PMC6353231; doi:10.1371/journal.pgen.1007808)
Supplement: S18 Table — (PDF) [file pgen.1007808.s020.pdf]

SNP	Chr.	Position (hg19)	A1	A2	OR	P-value	Prioritized genes
rs6670198	1	2520527	T	C	1.14	1.54E-36	MMEL1, TNFRSF14, TTC34, UBE2D3, C1orf93
rs2986736	1	6512547	C	T	1.12	8.91E-17	TNFRSF25
rs1801133	1	11856378	G	A	1.11	2.31E-15	MTHFR, MFN2
rs67934705	1	24207504	G	A	1.07	4.36E-08	HMGCL
rs6672420	1	25291010	A	T	1.06	1.48E-09	RUNX3, SYF2
chr1:32738415	1	32738415	A	G	1.13	1.44E-15	LCK,F AM167B
rs72922276	1	65429319	G	A	1.16	1.41E-15	JAK1, AK4, RAVER2
rs11161550	1	85682020	G	A	1.06	2.06E-09	BCL10
rs35486093	1	85729820	G	A	1.21	2.27E-31	LOC646626, BCL10, C1orf52
rs12133753	1	92222089	C	T	1.13	1.67E-16	TGFBR3
rs58394161	1	92939959	C	T	1.13	1.37E-14	
rs11809700	1	93152635	T	C	1.13	2.95E-30	EVI5, FAM69A
rs1415069	1	93426869	G	C	1.07	3.32E-08	FAM69A, GFI1, SNORA66, EVI5
rs34723276	1	101290432	A	G	1.10	1.11E-22	SLC30A7, DPH6
rs11578655	1	101412902	G	T	1.09	2.89E-08	SLC30A7
rs10801908	1	117090493	C	T	1.30	4.55E-70	CD58
rs483180	1	120267505	C	G	1.09	1.45E-17	PHGDH
chr1:154983036	1	154983036	G	T	1.18	1.67E-08	SHC1, KCNN3, ADAM15
rs2317231	1	157686337	G	T	1.08	3.25E-16	FCRL3, FCRL1, CD5L, FCRL2
rs3737798	1	160389984	A	G	1.07	4.12E-14	VANGL2, CASQ1, CD48
rs6427540	1	160634588	C	T	1.11	1.48E-09	SLAMF1
rs983494	1	160703965	G	A	1.09	6.10E-16	SLAMF7
rs1323292	1	192541021	A	G	1.16	3.64E-33	RGS1
rs59655222	1	200875897	T	C	1.10	5.66E-21	KIF21B, C1orf106
rs9308424	1	212877776	G	A	1.09	9.25E-14	BATF3, FLVCR1, UBE2D3

rs11899404	2	12607893	C	T	1.05	1.66E-08	MIR4262, TRIB2
rs11125803	2	25052177	C	T	1.07	5.45E-10	ADCY3, NCOA1
rs13414105	2	30472442	C	A	1.09	6.90E-10	LBH
rs12478539	2	43355324	G	C	1.12	1.65E-24	HAAO, ZFP36L2
rs1177228	2	61242410	G	A	1.10	2.31E-19	PUS10, AHSA2, C2orf74, USP34, KIAA1841, REL
rs13385171	2	65661843	C	T	1.07	3.47E-11	ACTR2, MIR4778, SPRED2
rs12622670	2	68646536	T	C	1.09	5.08E-16	PLEK, FBXO48
chr2:112492986	2	112492986	C	T	1.07	1.78E-12	ANAPC1
rs57116599	2	112770799	G	A	1.09	1.11E-11	MERTK, TMEM87B, ANAPC1
rs10191360	2	136884679	T	C	1.08	3.85E-10	CXCR4, THSD7B, DARS
rs962052	2	151644203	C	T	1.06	8.28E-09	RBM43, RND3
rs6738544	2	191989356	C	A	1.07	2.40E-13	STAT4
rs12614091	2	204632861	A	T	1.08	6.18E-12	CD28
rs35540610	2	231121829	C	T	1.14	2.98E-33	SP140, SP110
rs9863496	3	18798848	C	T	1.08	2.75E-14	KCNH8, SATB1
rs13327021	3	27783015	T	C	1.08	1.55E-16	EOMES
rs438613	3	28072086	C	T	1.15	2.31E-49	CMC1, EOMES
rs11919880	3	32962051	A	G	1.06	7.63E-10	CCR4, GLB1
rs9878602	3	71535338	T	G	1.10	1.91E-18	FOXP1
chr3:100848597	3	100848597	C	T	1.16	6.42E-11	NFKBIZ, PCNP, SENP7
rs4325907	3	101749022	C	T	1.08	1.99E-09	LOC152225, ZPLD1, SENP7
rs2289746	3	105455955	C	T	1.08	4.59E-12	CBLB
chr3:112693983	3	112693983	T	G	1.36	3.24E-09	CD200R1
rs9843355	3	119228508	G	A	1.15	4.14E-30	ARHGAP31, TMEM39A, CD80, POGLUT1
rs2331964	3	121542898	C	T	1.09	4.91E-20	IQCB1, EAF2, SLC15A2, HCLS1
chr3:121765368	3	121765368	C	G	1.14	6.98E-43	IQCB1
chr3:121783015	3	121783015	T	G	1.12	8.32E-10	

rs6789653	3	141150990	G	A	1.06	1.30E-09	ZBTB38, RASA2
rs1014486	3	159691112	C	T	1.11	3.13E-28	LINC01100, IL12A
rs10936182	3	159712373	T	G	1.10	4.99E-08	IL12A
rs10936602	3	169536637	T	C	1.08	1.90E-11	MYNN, LRRC34, LRRIQ4, LRRC31
rs2590438	3	187565968	G	T	1.07	4.65E-09	BCL6
rs13066789	3	187987624	C	T	1.07	1.50E-08	LPP
rs13136820	4	40307564	C	T	1.08	7.79E-10	
rs6837324	4	48127262	G	A	1.07	1.34E-11	TXK, TEC
rs2705616	4	87862396	C	G	1.08	1.15E-11	AFF1, LOC100506746
rs6533052	4	103911781	A	G	1.09	7.03E-16	MANBA, BDH2, CENPE, CISD2, NFKB1, SLC9B2, UBE2D3
rs2726479	4	106255589	C	T	1.07	3.84E-11	PPA2, TET2
rs9992763	4	109058718	G	T	1.07	2.36E-11	LEF1, RPL34-AS1
rs17051321	4	122119449	T	C	1.08	1.41E-10	NDNF, TNIP3
rs4361438	4	157575265	T	C	1.84	1.89E-09	
rs72989863	4	164493807	G	A	1.07	5.55E-09	MARCH1
rs34681760	5	6712834	C	T	1.08	2.01E-11	PAPD7
rs10063294	5	35877505	G	A	1.13	1.58E-28	IL7R, CAPSL
rs11749040	5	40396425	A	G	1.15	4.54E-25	DAB2, PTGER4
chr5:40429250	5	40429250	T	A	1.07	1.02E-11	
rs7731626	5	55444683	G	A	1.09	3.89E-15	ANKRD55, IL6ST
rs32658	5	118703662	T	G	1.06	3.24E-08	HSD17B4, TNFAIP8
rs244656	5	133449827	A	T	1.11	2.96E-14	TCF7
rs2084007	5	133891282	C	T	1.08	1.91E-13	CAMLG, JADE2, TCF7, PHF15
rs249677	5	141539339	A	C	1.07	5.73E-13	NDFIP1
rs2546890	5	158759900	A	G	1.09	5.30E-19	LOC285626, IL12B
rs67111717	5	176790162	G	A	1.10	3.18E-21	MXD3, RGS14, LMAN2
rs12211604	6	7100029	G	A	1.07	2.15E-08	RREB1

chr6:14691215	6	14691215	C	T	1.11	1.38E-17	CD83, JARID2
rs719316	6	16672760	T	C	1.07	1.62E-13	ATXN1
rs1076928	6	36348689	T	C	1.13	2.75E-19	PXT1, ETV7, KCTD20, STK38
rs72928038	6	90976768	A	G	1.15	8.38E-29	BACH2
chr6:119215402	6	119215402	A	C	1.08	3.24E-10	ASF1A, CEP85L, FAM184A, MCM9
rs802730	6	128280104	T	C	1.07	1.08E-09	PTPRK
rs2327586	6	135495226	T	C	1.11	9.48E-20	ALDH8A1, HBS1L, MYB
rs4896153	6	135833463	T	A	1.12	2.72E-29	AHI1, MYB
rs62420820	6	137438057	A	G	1.14	9.26E-36	IL20RA, IL22RA2
rs631204	6	137959455	A	C	1.11	4.92E-25	
rs17780048	6	138179146	C	T	1.10	5.15E-12	TNFAIP3
rs6911131	6	143865221	G	A	1.12	1.31E-10	ADAT2, FUCA2, LOC285740, PHACTR2
rs1738074	6	159465977	C	T	1.13	3.48E-35	TAGAP, RSPH3
rs55858457	7	2443302	T	G	1.07	2.95E-08	EIF3B, CHST12, CARD11, SDK1
rs10951042	7	3139417	C	T	1.10	1.50E-18	CARD11
rs10951154	7	27135314	C	T	1.08	3.05E-10	HOXA1
rs10245867	7	28142186	T	G	1.07	1.22E-11	JAZF1, JAZF1-AS1
rs60600003	7	37382465	G	T	1.15	4.60E-19	ELMO1
rs10230723	7	50239880	A	T	1.09	7.69E-11	
chr7:50328339	7	50328339	A	G	1.09	2.58E-16	C7orf72, IKZF1
rs73414214	7	105706462	C	A	1.13	6.34E-10	SYPL1
rs4728142	7	128573967	A	G	1.06	3.37E-09	IRF5, TNPO3, KCP
rs10271373	7	138729795	A	C	1.06	3.11E-09	ZC3HAV1, ZC3HAV1L
rs354033	7	149289464	G	A	1.08	7.99E-10	ZNF746
rs28703878	8	79417222	G	A	1.11	5.27E-22	ZC2HC1A, PKIA
chr8:95851818	8	95851818	G	T	1.15	3.23E-10	TP53INP1, INTS8
rs735542	8	128175696	A	G	1.07	5.66E-13	

rs6990534	8	128814091	G	A	1.10	5.85E-20	LINC00977, MIR1208, PCAT1, POU5F1B, PVT1
chr8:129177769	8	129177769	C	T	1.12	2.62E-25	
rs3923387	8	144986793	T	C	1.06	1.76E-08	PLEC, PARP10, SPATC1, MAF1, GRINA
rs7855251	9	100868189	T	C	1.08	1.23E-10	NANS, C9orf156, TRIM14
rs12722559	10	6070273	C	A	1.14	1.76E-15	
rs11256593	10	6117322	T	C	1.21	2.96E-65	IL2RA, IL15RA
rs1399180	10	8098719	C	T	1.10	7.16E-11	GATA3
rs1087056	10	31395761	A	G	1.09	3.50E-19	ZEB1-AS1, ZNF438
rs61863928	10	64449549	G	T	1.09	3.01E-16	ADO
rs17741873	10	75653800	G	T	1.07	4.69E-08	PLAU, NDST2, KIAA0913, CAMK2G
rs1250551	10	81059335	T	G	1.10	1.68E-23	ZMIZ1
rs1112718	10	94479107	A	G	1.09	2.08E-17	HHEX, IDE
rs35218683	11	321138	T	C	1.08	1.36E-08	IFITM3
rs61884005	11	14402930	C	G	1.10	6.34E-09	RRAS2, PDE3B
chr11:14868316	11	14868316	G	A	1.29	8.76E-12	CYP2R1
rs1365120	11	36438075	C	T	1.10	4.06E-08	PRR5L
rs2269434	11	47360412	C	T	1.09	1.47E-13	ACP2, MADD, MYBPC3, RAPSN, DDB2, MTCH2, PSMC3, SPI1, NR1H3
rs4939490	11	60793651	G	C	1.12	2.00E-29	CD6, CD5
rs11231749	11	64095178	C	T	1.07	1.12E-11	VEGFB, PLCB3, CCDC88B, RPS6KA4, FKBP2, GPR137
rs531612	11	65705432	T	C	1.07	1.21E-09	EFEMP2,FOSL1,TSGA10IP,SART1,CTSW,SNX32,FIBP,BANF1,CCDC85B,CST6,SF3B2,C11orf68
rs4409785	11	95311422	C	T	1.09	6.87E-12	SESN3,FAM76B
rs56095240	11	95421830	A	T	1.11	1.13E-09	
rs34026809	11	118480695	G	C	1.09	7.68E-17	TMEM25,DDX6,CXCR5
rs12365699	11	118743286	G	A	1.12	2.10E-19	TMEM25,DDX6,CXCR5
rs6589706	11	118747813	A	G	1.11	4.53E-26	DDX6,TMEM25,CXCR5
chr11:118783424	11	118783424	G	A	1.27	8.76E-11	MLL
rs6589939	11	122518525	G	A	1.08	1.75E-12	UBASH3B

rs4262739	11	128421175	G	A	1.07	4.41E-12	ETS1
rs1800693	12	6440009	C	T	1.15	2.24E-47	TNFRSF1A,U47924.1
rs12832171	12	6441622	C	G	1.17	4.13E-10	TNFRSF1A
rs2364485	12	6514963	A	C	1.12	1.51E-20	LTBR
rs7977720	12	9866349	T	C	1.10	4.96E-24	CLEC2D,CLECL1
rs701006	12	58106836	G	A	1.12	9.63E-31	OS9,METTL21B,TSFM,XRCC6BP1,TSPAN31,CYP27B1,MARCH9,B4GALNT1
rs61708525	12	94661453	G	A	1.07	1.80E-08	PLXNC1
rs3184504	12	111884608	T	C	1.06	4.24E-11	SH2B3,CUX2,ALDH2,HECTD4,TRAFD1
rs7975763	12	123604053	T	C	1.09	2.99E-13	PITPNM2,MPHOSPH9,SBNO1,CDK2AP1,OGFOD2,SETD8,SNRNP35,C12orf65,RILPL2
rs9591325	13	50811220	T	C	1.23	1.26E-19	DLEU7,ST13P4
rs9568402	13	50961957	T	A	1.09	4.80E-09	
chr13:100026952	13	100026952	A	C	1.11	8.98E-17	MIR548AN,TM9SF2
rs11852059	14	52306091	C	A	1.10	1.19E-09	FRMD6,GNG2
rs12434551	14	69253364	A	T	1.08	4.14E-17	ZFP36L1
rs34695601	14	76014298	T	C	1.08	5.56E-13	BATF,FLVCR2
chr14:88523488	14	88523488	C	T	1.33	1.67E-30	GALC
rs12588969	14	103230758	G	C	1.08	1.82E-09	TRAF3
rs12147246	14	103265844	A	G	1.09	1.98E-15	TRAF3
rs62013236	15	79247482	C	T	1.11	8.53E-17	CTSH
rs6496663	15	90887584	C	A	1.09	1.13E-12	IQGAP1
rs405343	16	1067832	T	G	1.10	3.05E-15	SOX8,SSTR5-AS1
rs2286974	16	11114512	A	G	1.10	4.09E-09	CLEC16A,DEXI
chr16:11213951	16	11213951	C	T	1.20	3.70E-71	DEXI,CLEC16A
chr16:11353879	16	11353879	T	C	1.09	5.01E-16	RMI2
rs34947566	16	11412926	C	A	1.15	1.30E-23	RMI2,CLEC16A
rs3809627	16	30103160	C	A	1.09	1.23E-21	FAM57B,TBX6,GDPD3,INO80E,MAPK3,PPP4C,TAOK2,YPEL3,RP11-345J4.5,SEPT1
rs8062446	16	57077094	T	C	1.09	4.30E-09	NLRC5

rs12925972	16	79111297	C	T	1.11	1.11E-19	DYNLRB2,WWOX
rs17724508	16	79350204	T	C	1.14	6.07E-14	
rs6564681	16	79652720	C	T	1.08	3.70E-14	
rs35703946	16	86021505	G	A	1.13	2.83E-17	IRF8,LINC01082
rs4796224	17	34842521	G	A	1.09	1.13E-15	MYO19,GGNBP2,DHRS11,MRM1,PIGW
rs9909593	17	37970149	G	A	1.08	8.57E-17	IKZF3,ZPBP2,GSDMB,ORMDL3,PGAP3,GSDMA
rs883871	17	38252660	A	G	1.13	8.56E-14	THRA,NR1D1
rs1026916	17	40529835	A	G	1.11	2.32E-28	STAT3
rs7222450	17	43407670	A	G	1.06	2.09E-10	MAP3K14
rs11079784	17	45702280	C	T	1.11	1.99E-27	EFCAB13,TBKBP1,C17orf57,NPEPPS
rs2150879	17	57859210	G	A	1.12	4.05E-31	TUBD1,RNFT1,HEATR6,BCAS3,VMP1,MIR21
rs9900529	17	73335776	C	G	1.06	4.76E-08	SLC25A19,GRB2,MIF4GD,MRPS7,NUP85,CASKIN2,MYO15B,GGA3,TSEN54
rs4940730	18	56269737	A	G	1.06	3.90E-10	ALPK2
rs9955954	18	56348044	A	G	1.09	4.85E-11	MALT1
rs2469434	18	67544046	C	T	1.05	2.92E-08	CD226
rs12971909	19	4466466	A	G	1.07	5.56E-09	CHAF1A,CTB-50L17.10,UBXN6,HDGFRP2
rs1077667	19	6668972	C	T	1.15	7.88E-33	TNFSF14,C3
rs34536443	19	10463118	G	C	1.20	3.39E-11	TYK2,ICAM3
rs28834106	19	10592144	T	C	1.12	3.61E-19	PDE4A,KEAP1,KRI1,SMARCA4
rs12609500	19	11173928	C	T	1.06	5.27E-09	SMARCA4,ATG4D
rs58166386	19	16559421	G	A	1.11	4.42E-24	CALR3,C19orf44,EPS15L1
rs4808760	19	18301979	C	G	1.12	5.83E-25	IFI30,MPV17L2,PDE4C,KIAA1683,MAST3
rs7260482	19	45143942	C	A	1.08	1.43E-09	PVR
rs11083862	19	47638539	A	T	1.08	2.44E-09	HIF3A
rs1465697	19	49837246	T	C	1.10	3.02E-18	DKKL1,CD37,TEAD2,SLC6A16
rs4812492	20	39938122	C	T	1.07	2.83E-06	LPIN3,PLCG1,TOP1
rs4812772	20	42579051	C	T	1.07	6.17E-09	TOX2

rs6032662	20	44734310	C	T	1.10	5.28E-19	SLC12A5,CD40,NCOA5
rs6020055	20	48422095	A	G	1.06	5.12E-08	B4GALT5,SLC9A8
rs2585447	20	52744437	C	T	1.09	3.13E-10	
rs2248137	20	52789743	C	G	1.09	1.92E-19	CYP24A1,BCAS1
rs6742	20	62374441	C	T	1.15	4.11E-14	ZGPAT
rs9808753	21	34787312	G	A	1.08	1.60E-09	IFNGR2,TMEM50B,ITSN1
rs2836438	21	39864727	A	G	1.11	2.62E-10	TMPRSS3,ERG
rs9610458	22	22205353	T	C	1.09	1.16E-19	TOP3B,PPM1F,MAPK1
rs4820955	22	31622539	A	T	1.07	1.06E-08	PIK3IP1,PISD,SFI1,LIMK2
rs760517	22	37258986	C	T	1.07	5.22E-11	NCF4,PVALB,CSF2RB
rs5756405	22	37310954	A	G	1.06	5.35E-11	NCF4,CSF2RB
rs137955	22	40291807	T	C	1.06	9.63E-09	GRAP2,ENTHD1
rs140522	22	50971266	T	C	1.12	1.31E-21	SCO2,TYMP,CHKB,NCAPH2,CPT1B,ODF3B
rs2807267	X	135666453	T	G	1.07	$6.86x10^{-9}$	RNU6-320P
